# Effect of occupational therapy on upper limb function and related rehabilitation outcomes after stroke: a systematic review and meta-analysis

**DOI:** 10.3389/fneur.2026.1692088

**Published:** 2026-04-23

**Authors:** Jie Bai, Tianyun Liu, Xuelian Du, Wenrui Huang

**Affiliations:** 1 Shenzhen Polytechnic University, Shenzhen, Guangdong, China; 2 The First Affiliated Hospital of Tianjin University of Traditional Chinese Medicine, Tianjin, China; 3 Shenzhen Traditional Chinese Medicine Hospital, Shenzhen, Guangdong, China

**Keywords:** activities of daily living, meta-analysis, occupational therapy, stroke rehabilitation, upper limb function

## Abstract

**Background:**

Stroke is a major cause of disability worldwide, often leading to upper limb dysfunction, reduced daily living ability, and depression. Occupational therapy (OT) is recommended in rehabilitation guidelines, yet evidence on its comprehensive effects across physical and psychological outcomes in stroke survivors remains limited.

**Objective:**

This systematic review and meta-analysis aimed to provide the most up-to-date and comprehensive evaluation of the effects of OT on upper limb function, daily living ability, and depressive symptoms in stroke patients.

**Method:**

Eight English and Chinese databases were searched to August 2025 for RCTs comparing OT with standard rehabilitation in stroke patients. Outcomes included upper limb function, ADL, and depressive symptoms. Risk of bias was assessed with the Cochrane tool, and certainty of evidence with GRADE. Meta-analyses were conducted in RevMan 5.4 and Stata 18.0, with sensitivity and subgroup analyses by intervention setting, OT type, and duration. The complete search strategies (full electronic search strings for each database) are provided in Appendix 2.

**Results:**

Twenty-two RCTs involving 2,833 stroke patients were included. Meta-analysis showed that OT significantly improved upper limb function (SMD = 1.42, 95% CI 0.62–2.21, *p* = 0.0005), activities of daily living (SMD = 1.17, 95% CI 0.80–1.54, *p* < 0.00001), and reduced depressive symptoms (SMD = −2.08, 95% CI –3.01 to −1.15, *p* < 0.00001) compared with controls. Subgroup analyses suggested larger effects in certain settings, with specific intervention types, and with longer (>8 weeks) durations, particularly for motor recovery. Sensitivity analyses confirmed result stability, and GRADE certainty was rated moderate for all outcomes.

**Conclusion:**

OT significantly improves upper limb function, daily living ability, and depressive symptoms in stroke patients, with greater gains seen in longer-term and structured programs, supporting its integration into post-stroke rehabilitation.

**Systematic review registration:**

https://www.crd.york.ac.uk/PROSPERO/, identifier CRD420251122701.

## Introduction

Stroke refers to a sudden disruption of blood flow to the brain, leading to neurological impairment. It is the second leading cause of death worldwide and the primary cause of death and disability among Chinese adults (1). In 2019, an estimated 12.2 million people experienced a stroke globally, resulting in 6.55 million deaths (2). That same year, China reported approximately 3.94 million new stroke cases and 2.19 million related deaths (3). The consequences of stroke are often long-lasting and severe. Most patients develop motor impairments, particularly in the upper limbs, such as weakness and poor coordination. These deficits significantly limit their ability to perform daily activities and reduce overall quality of life (4). In addition, physical disability often leads to psychological challenges, including depression and emotional distress, which further hinder recovery (5).

Rehabilitation is central to stroke management. Chinese clinical guidelines recommend limb-focused therapies, including joint mobility exercises, strength training, and gait rehabilitation (6). They also acknowledge the American Heart Association’s support for incorporating occupational therapy (OT) into post-stroke care. For the purposes of this review, OT is operationally defined as any structured, goal-oriented intervention delivered by a qualified occupational therapist, encompassing activities of daily living (ADL) training, task-specific upper limb practice, interest-based or group therapy, domiciliary OT, and device-mediated approaches, but excluding interventions primarily classified as physiotherapy or mixed rehabilitation without a distinct occupational therapy component. Occupational therapy helps patients regain independence through goal-oriented activities drawn from everyday life—such as self-care, work, and leisure—tailored to their individual needs (7). Common interventions include training for daily living tasks, upper limb coordination, and fine motor skills. OT follows a stepwise, personalized approach, adjusting to each patient’s progress. This flexibility promotes engagement, supports better recovery outcomes, and may shorten rehabilitation time (8).

Although earlier systematic reviews (9, 10) demonstrated beneficial effects of OT on ADL outcomes after stroke, these reviews are now over two decades old, neither comprehensively addressed upper limb motor function as a primary outcome, nor examined the impact of OT on depressive symptoms. The present study therefore conducts an updated systematic review and meta-analysis of randomized controlled trials to address these gaps, with upper limb motor function and ADL performance as primary outcomes and depressive symptoms as a secondary outcome. The findings aim to inform clinical decision-making and support the development of more effective rehabilitation strategies. We hypothesized that, compared with standard care or conventional rehabilitation alone, OT would significantly improve upper limb function, ADL, and depressive symptoms in adults with stroke.

## Methods

This systematic review and meta-analysis was conducted in accordance with the Preferred Reporting Items for Systematic Reviews and Meta-Analyses (PRISMA) 2020 guidelines (Appendix 1) (11). The protocol for this review was prospectively registered in the International Prospective Register of Systematic Reviews (PROSPERO; registration number: CRD420251122701).

### Data sources and search strategy

A comprehensive literature search was conducted across eight databases: China National Knowledge Infrastructure (CNKI), Wanfang Data, Weipu (VIP) Database, Chinese Biomedical Literature Service System (SinoMed), Web of Science, Cochrane Library, PubMed, and EMBASE. The search included all records from database inception to August 2025. Where available, “ahead of print” and “in-press” records indexed within the searched databases up to the search date were also screened for eligibility. Search terms included a combination of keywords and MeSH terms such as “occupational therapy,” “OT,” “stroke,” “cerebral apoplexy,” “stroke rehabilitation,” “post-stroke,” and “randomized.” The detailed PubMed search strategy is presented in Appendix 2. To capture gray literature and ongoing/unpublished trials, we also searched trial registries (ClinicalTrials.gov and the WHO International Clinical Trials Registry Platform [ICTRP]) and screened conference proceedings and relevant clinical practice guidelines. No eligible unpublished or gray literature records meeting our inclusion criteria were identified through these searches. In addition, reference lists of included studies and relevant reviews were hand-searched to identify additional eligible RCTs. Records identified from registries and gray literature were screened using the same eligibility criteria as published studies; when necessary, investigators were contacted for available outcome data.

### Inclusion and exclusion criteria

Eligible studies were randomized controlled trials (RCTs) published in English or Chinese. Eligibility criteria were prespecified according to the PICOS framework:

Population (P): Adults diagnosed with stroke confirmed by neuroimaging (CT or MRI), including ischemic stroke and/or intracerebral hemorrhage. We included patients across stages of chronicity (acute, subacute, or chronic) as defined by the original trials; when not explicitly stated, stage was categorized using the time since stroke onset reported in the study. Baseline stroke severity and upper-limb impairment were extracted where available (e.g., NIHSS, Fugl–Meyer baseline score, Brunnstrom stage, or equivalent clinical scales).

Intervention (I): OT operationally defined as a goal-directed, therapist-led rehabilitation program primarily targeting upper-limb motor recovery and/or performance of activities of daily living (ADL) through structured task practice. OT interventions were eligible if they included at least one of the following core components: (1) task-oriented upper-limb training (repetitive practice of functional tasks); (2) ADL-based training (self-care, dressing, feeding, grooming, transfers, and/or instrumental ADL practice); (3) constraint-induced movement therapy (CIMT) or modified CIMT delivered within an OT framework; (4) cognitive–motor OT integrating cognitive strategies (e.g., attention/executive training) with upper-limb or ADL task practice; and/or (5) home-based OT with structured, therapist-prescribed activities and progression. Studies were excluded if “OT” was not clearly described, was indistinguishable from general physiotherapy, or consisted solely of non-specific exercise without task/ADL content. When the nature of an intervention was ambiguous during screening-for example, when a study described a combined or multidisciplinary program without clearly delineating the OT component-the two independent reviewers discussed the case with reference to the operational definition above. If the intervention could not be confirmed as primarily OT-led (i.e., with OT as the principal and distinct therapeutic component rather than an adjunct to physiotherapy), the study was excluded. Disagreements were resolved by a third reviewer.

Comparator (C): Standard rehabilitation (usual care), operationally defined as conventional post-stroke rehabilitation programs not explicitly incorporating structured OT components, such as routine physiotherapy/exercise therapy (range-of-motion, strengthening, balance, gait training), basic nursing care, and/or general health education. When OT was delivered in addition to usual care, the comparator was required to receive the same usual care intensity without the OT-specific components.

Outcomes (O): Studies reporting at least one prespecified outcome: (1) upper-limb function (e.g., Fugl–Meyer Assessment–Upper Extremity, Action Research Arm Test, Wolf Motor Function Test, Box and Block Test, or equivalent); (2) ADL (e.g., Barthel Index, Functional Independence Measure, Modified Barthel Index, or equivalent); and/or (3) depressive symptoms (e.g., Hamilton Depression Rating Scale, Beck Depression Inventory, Patient Health Questionnaire-9, or equivalent). A summary of the specific outcome measurement tools used in each included study, organized by outcome domain, is provided in Appendix 3.

Study design (S): Parallel-group RCTs. Cluster RCTs were eligible if appropriate statistical adjustment was reported or could be derived.

Studies were excluded if they were duplicate publications (the most complete or recent report was retained), conference abstracts without full text, non-randomized designs, or had insufficient/irretrievable outcome data for effect size estimation.

### Data extraction and quality assessment

Two reviewers independently screened all retrieved records against the prespecified eligibility criteria, first at the title and abstract level and then at full text. Discrepancies at either stage were resolved through discussion with a third reviewer. From each included study, the following data were extracted using a standardized form: first author, publication year, country, sample size (intervention and control groups), participant mean age, menopausal status and definition, type and description of the DHI, duration and frequency of the intervention, delivery format (self-directed versus therapist-guided), behavior change techniques incorporated, comparator condition, outcome domains assessed, and measurement instruments used. Where multiple time points were reported, data from the primary endpoint were extracted as the main outcome; end-of-intervention and longest available follow-up data were also recorded separately to assess durability of effects.

Methodological quality of all included RCTs was assessed independently by two reviewers using the Cochrane Risk of Bias Tool (RoB 1), evaluating six domains: random sequence generation, allocation concealment, blinding of participants and personnel, blinding of outcome assessment, incomplete outcome data, selective outcome reporting, and other potential sources of bias. Each domain was rated as low risk, unclear risk, or high risk of bias. Given the inherent difficulty of blinding participants to a digital intervention, performance bias was interpreted with particular caution; studies were not downgraded solely on the basis of participant non-blinding where outcome assessment was conducted independently. Overall risk of bias profiles were used in sensitivity analyses to examine whether study quality moderated pooled effect estimates. Disagreements were resolved by consensus or adjudication by a third reviewer.

### Certainty of evidence assessment

The quality of evidence for each outcome was independently evaluated using the GRADE (Grading of Recommendations, Assessment, Development and Evaluation) framework, which is widely applied in systematic reviews involving randomized controlled trials. This assessment considered five key factors: potential risk of bias, heterogeneity of results, relevance of the evidence to the research question, precision of the estimates, and the likelihood of publication bias.

### Statistical analysis

All statistical analyses were performed using RevMan version 5.4 and Stata version 18.0. For continuous outcomes, data were pooled based on the comparability of measurement methods and instruments. When outcome measures were assessed using the same scale and tools, the mean difference (MD) was used as the effect estimate. In cases where different measurement tools were employed, the standardized mean difference (SMD) was applied. The use of SMD is clinically justified when pooling heterogeneous instruments within the same outcome domain, as SMD expresses each study’s effect relative to its own observed variability, thereby placing results from different scales on a common metric while preserving the direction and magnitude of the treatment effect. All instruments pooled within each domain (upper limb function, ADL, and depressive symptoms) measure the same underlying clinical construct, supporting the appropriateness of this approach. All effect estimates were reported with their corresponding 95% confidence intervals (CIs). For studies reporting outcomes at multiple post-treatment time points, the final assessment time point (i.e., the longest available follow-up) was selected for the primary analysis, as this best reflects the sustained effects of OT. Where studies reported only a single post-treatment time point, that measurement was used. This approach was applied consistently across all included studies. Heterogeneity across studies was assessed using the Chi-square (*χ*^2^) test and the *I*^2^ statistic. A fixed-effects model was applied when heterogeneity was low (*I*^2^ < 50%). In the presence of significant heterogeneity (*I*^2^ > 50%), a random-effects model was used. If substantial heterogeneity was identified, sensitivity analyses were conducted to explore potential sources. Publication bias was evaluated visually using funnel plots. Subgroup analyses were performed to further investigate potential sources of heterogeneity and to assess the robustness of the findings, stratifying studies according to intervention settings, OT intervention type, and intervention duration.

## Results

### Literature screening and included studies

**Figure 1:**
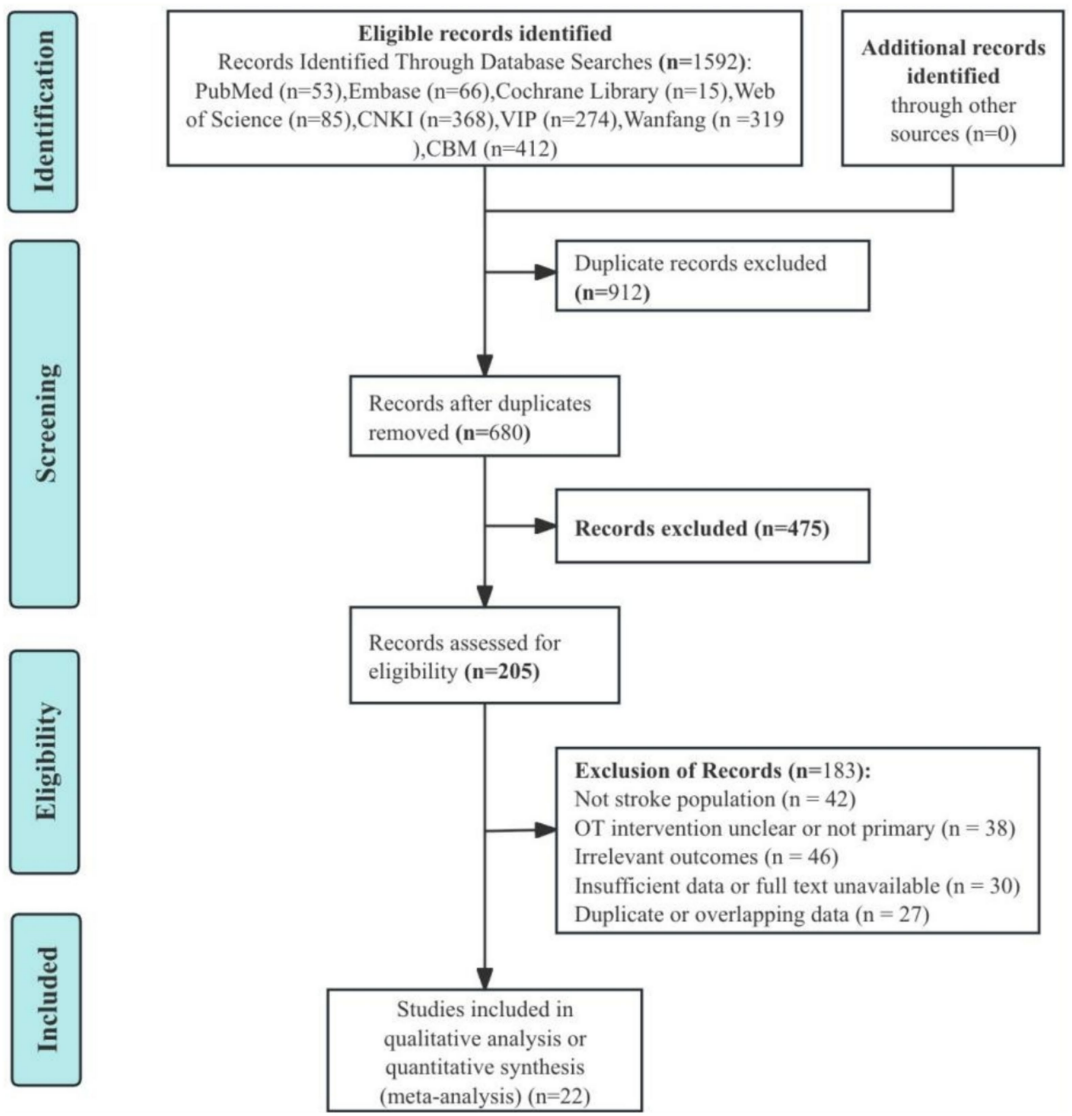
Flowchart showing study retrieval and inclusion.

A total of 1,592 records were initially identified through systematic searches of eight databases, including PubMed (*n* = 53), Embase (*n* = 66), Cochrane Library (*n* = 15), Web of Science (*n* = 85), CNKI (*n* = 368), VIP (*n* = 274), Wanfang (*n* = 319), and CBM (*n* = 412). After removing 912 duplicates, 680 records remained for title and abstract screening. Of these, 475 records were excluded due to irrelevance. The full texts of 205 articles were assessed for eligibility. A total of 183 articles were excluded for the following reasons: not stroke population (*n* = 42), OT intervention unclear or not primary (*n* = 38), irrelevant outcomes (*n* = 46), insufficient data or full text unavailable (*n* = 30), and duplicate or overlapping data (*n* = 27). Finally, 22 studies (12–33) were included in this meta-analysis. The study selection process is presented in the PRISMA flow diagram (Figure 1).

### Characteristics of included studies

A total of 22 randomized controlled trials were included in this meta-analysis, comprising 2,833 patients—1,500 in the intervention groups and 1,333 in the control groups. The sample sizes of individual studies ranged from 30 to 942 participants. The studies were conducted between 2000 and 2022 and were published in both Chinese and English. The mean age of participants across studies varied widely, ranging from approximately 50 to over 80 years. Most studies reported comparable baseline age distributions between intervention and control groups. Treatment durations also varied considerably, from as short as 2 weeks to as long as 6 months. The majority of interventions lasted between 4 and 8 weeks, with a few studies reporting treatment frequencies (e.g., daily sessions or hours per session) rather than duration in weeks. Detailed characteristics of the included studies—including author, publication year, sample size, age, and intervention duration—are presented in Table 1.

**Table 1:** Baseline characteristics of included studies.

Study	N (T/C)	Age (T/C)	OT type (category)	OT core components (brief)	Dose/frequency (h/week when available)	Setting	Follow-up	Outcomes
Dong 2007 (12)	36/36	50.7 / NR	ADL-focused OT	ADL relearning; fine motor training; assistive devices	1×/day, 60 min (h/week NR)	Inpatient	6 w	①②
Chen 2018 (13)	60/60	56.8 ± 5.49/56.9 ± 5.13	Task-oriented + ADL OT	Upper-limb control; grasp practice; fine motor tasks; self-care	2×/day, 20–30 min (≈40–60 min/day; h/week NR)	Inpatient	4 w	①②
Cai 2018 (14)	40/39	58.3 ± 4.7/59.8 ± 4.9	ADL-focused OT	Fine motor practice; compensation training; assistive tools; ADL (feeding/dressing/toothbrushing)	1×/day, 60 min (h/week NR)	Inpatient	NR	①②
Zeng 2020 (15)	35/35	51.7 ± 3.2/53.4 ± 2.7	ADL-focused OT	ADL relearning (turning, dressing, feeding, transfers)	NR (with PT)	Inpatient	6 w	②③
Jing 2006 (16)	120/40	54.5 ± 19.6/57.3 ± 12.5	Staged OT (ADL + fine motor)	Early ADL relearning; later fine motor/selected tasks; assistive devices; compensation strategies	1×/day, 45–60 min (h/week NR)	Inpatient	7 w	①②
Xing 2015 (17)	40/40	54.42 ± 12.37/55.63 ± 12.51	Functional + vocational OT	Functional tasks; personal ADL; assistive devices; vocational activities (e.g., woodworking/weaving)	1×/day, 45 min (h/week NR)	Inpatient	4 w	②③
Qian 2007 (18)	52/50	65.38 ± 9.21/64.54 ± 9.46	Upper-limb + fine motor OT	Early grasp training; later fine motor devices/tasks; ADL-related practice	2×/day, 45 min, 5 d/wk. (7.5 h/wk)	Inpatient/Outpatient	2 w	①②
Jie 2018 (19)	34/34	57.24 ± 11.63/59.37 ± 12.81	Task-oriented + ADL OT	ROM/weight-bearing; grasp training; fine motor (putty/blocks); ADL practice	2×/day, 45 min, 5 d/wk. (7.5 h/wk); total duration NR	Inpatient	NR	②③
Di 2011 (20)	20/20	68.5 ± 7.1/69.1 ± 9.2	Upper-limb control + fine motor OT	ROM/control; fine motor boards; grasp/pinch; assembly tasks	NR (increased OT; session time NR)	Inpatient	8 w	①
Chen 2015 (21)	38/36	62.35/60.57	Home-based task-oriented OT	Task-based (table-wiping) with level-specific progression; ADL integration	3×/day, 40 min, ≥5 d/wk. (≥10 h/wk)	Home/Community	12 w	①②
Lai 2021 (22)	30/30	55.23 ± 8.41/55.46 ± 8.45	Ward-extension OT (ADL-integrated)	Routine OT + ward-integrated ADL practice; “affected-hand guided by unaffected hand”	Routine OT 45 min/day, 5 d/wk. (3.75 h/wk) + daily ward extension (time NR)	Inpatient (ward extension)	4 w	①②
Gu 2020 (23)	20/20	67.75 ± 9.34/67.42 ± 9.74	ADL + fine motor OT (±NDT)	ADL training; upper-limb control; neurodevelopmental facilitation (e.g., Bobath); fine motor tasks	45 min/day, 5 d/wk. (3.75 h/wk)	Inpatient	6 w	②
Jiang 2017 (24)	20/20	60.16 ± 10.75/59.54 ± 11.30	Interest-based/group OT	Music-based exercises; modified tasks; group games; social/leisure activities	2×/day, 1 h, 5 d/wk. (10 h/wk)	Inpatient	2 w	②③
Lin 2007 (25)	30/28	63.52 ± 7.1/61.14 ± 9.2	Staged OT + ADL OT	Phase-based training (flaccid/spastic/recovery); fine motor tasks; ADL drills	40 min/day, 5 d/wk. (3.33 h/wk)	Inpatient	4 w	①②
Akiyama 2021 (26)	15/15	64.4 ± 13.7/60.5 ± 13.8	Task-oriented OT (device-based)	Screw Block® 3D assembly (finger control) + routine OT	5 d/wk.: 20 min Screw Block + 20–40 min routine OT (3.3–5.0 h/wk)	Inpatient	3 w	①②
Aydilek 2022 (27)	25/25	63 (30–79) / 67 (49–80)	Upper-limb + ADL OT	Fine hand skills using materials; coordination; ADL	45 min, 3 d/wk. (2.25 h/wk)	Inpatient	6 w	①②
Eroğlu 2020 (28)	17/18	56.9 ± 11.0/59.7 ± 11.9	Individualized task-oriented OT	Personalized ADL-related fine tasks (beading, buttoning, games, putty, pegboard)	45 min, 3 d/wk. (2.25 h/wk)	Outpatient	8 w	①②③
Gilbertson 2000 (29)	67/71	71 (28–89) / 71 (31–89)	Client-centered home OT	Goal-based self-care/household/leisure activities	~10 visits, 30–45 min/visit (total ≈5–7.5 h over 6 w; ~0.8–1.3 h/wk)	Home	8 w	②
Parker 2001 (30)	153/157	72 ± 10.37/72 ± 9.62	Leisure OT vs. ADL OT	Leisure-focused OT vs. ADL-focused OT (e.g., cooking, mobility/self-care)	≥10 visits; ≥30 min/visit (avg ~ 50–60 min)	Community/Home	6 mo	②
Sackley 2006 (31)	63/55	88.6 ± 6.5/86.3 ± 8.8	Personal ADL-focused OT	Feeding, dressing, transfers, mobility; caregiver education; environmental modification	median 2.7 visits/mo; median 4.5 h/mo (~1.0 h/wk)	Care homes	6 mo	②
Sackley 2015 (32)	512/430	83.6 ± 9.5/83.1 ± 9.9	Goal-setting + ADL OT	ADL task-specific training; environmental adaptation; staff training	mean 5.1 visits total; median 30 min/visit (total ≈2.6 h over 3 mo; ~0.2 h/wk)	Care homes	6 mo	②③
Walker 2001 (33)	73/74	73.3 ± 7.8/74.7 ± 8.0	Home OT (ADL + extended ADL)	Promoting independence in personal ADL + extended ADL	1–15 visits (mean 6); dose per visit NR	Home	5 mo	②

T: occupational therapy (OT) group; C: control group. Outcomes: ① upper limb function, ② activities of daily living (ADL), ③ depressive symptoms. w = weeks; m = months; NA = not reported.

### Intervention characteristics

The included studies implemented a wide range of OT interventions, reflecting variations in clinical settings, treatment intensity, and therapy content. Most OT protocols were delivered in combination with standard physical therapy (PT), while several studies evaluated OT as a stand-alone intervention. The duration of OT interventions ranged from 2 weeks to 6 months, with session frequencies varying between once daily and multiple times per day, typically lasting 30–60 min per session. Common OT components included training in ADLs, upper limb functional exercises, fine motor skill development, task-oriented activities, use of assistive devices, and patient education. Some studies emphasized individualized, goal-directed therapy based on patient needs and recovery stages, while others incorporated group-based or home-based training. A few trials integrated novel approaches such as game-based tasks, music-assisted activities, or tool-based interventions (e.g., pegboards, therapy boards). Control groups generally received conventional rehabilitation, including physical therapy or standard post-stroke care, without targeted OT. A subset of studies also included psychological support, caregiver involvement, or structured education as part of the OT or control protocols. Further details are provided in Appendix 3.

### Risk of bias assessment

**Figure 2:**
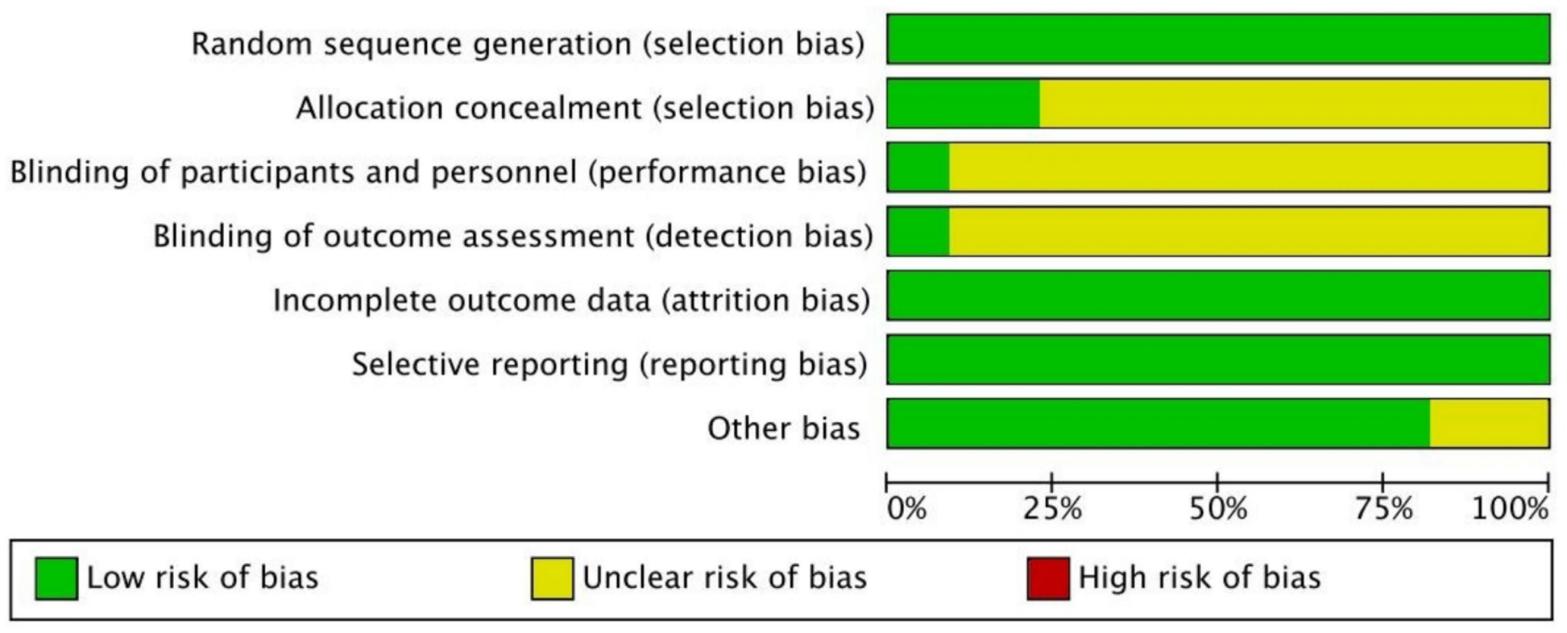
Risk of bias graph.

**Figure 3:**
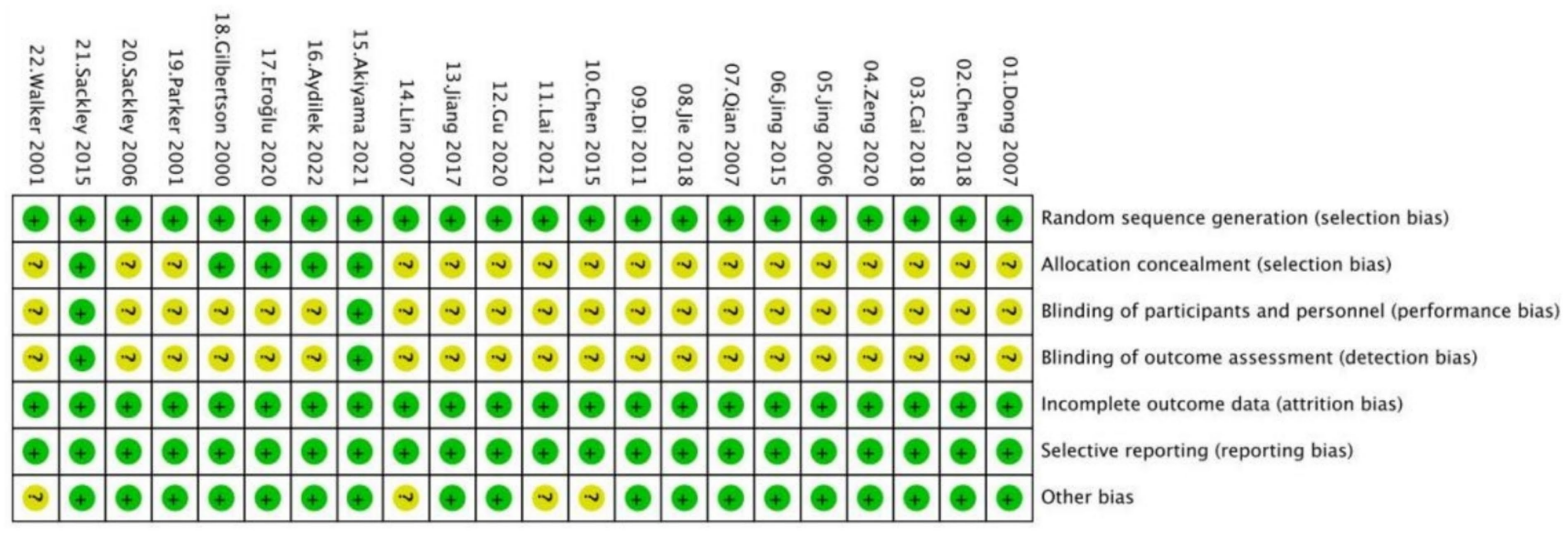
Risk of bias summary.

Most of the included studies showed a low risk of bias in handling missing outcome data and in reporting results, indicating generally reliable follow-up and transparent reporting practices. All studies were rated as low risk in these two domains. Random sequence generation was adequately reported in the majority of studies, suggesting appropriate randomization. However, the risk of bias related to allocation concealment, blinding of participants and personnel, and blinding of outcome assessment was unclear in most cases. This was largely due to a lack of detailed reporting on these methods. As a result, concerns remain about potential selection, performance, and detection bias in these areas. A summary of overall risk of bias is presented in Figure 2, with detailed assessments for each study shown in Figure 3.

### Upper limb function

**Figure 4:**
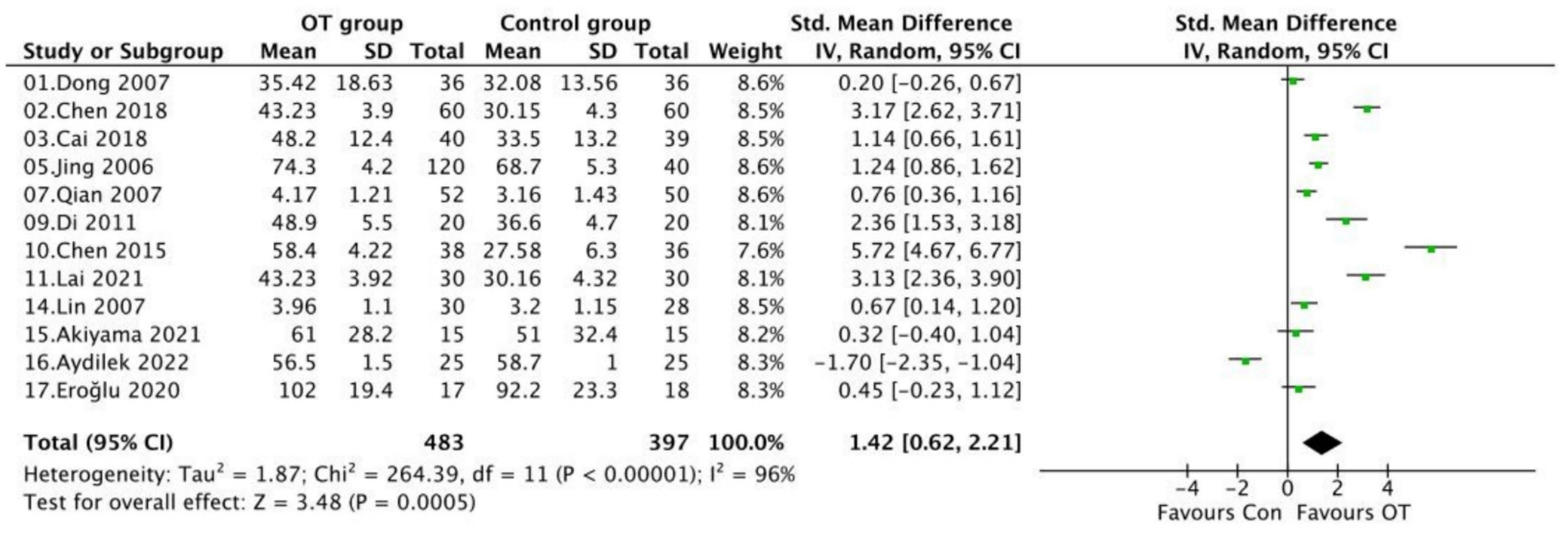
Forest plot comparing upper limb function between the OT and control group.

Twelve studies evaluated the effects of occupational therapy on upper limb function in stroke patients. Due to high heterogeneity (*I*^2^ = 96%, *p* < 0.00001), a random-effects model was applied. Pooled results showed that occupational therapy significantly improved upper limb function compared with control interventions (SMD = 1.42, 95% CI [0.62, 2.21], *p* = 0.0005; Figure 4). Most studies favored the OT group, with several (e.g., Chen 2018, Jing 2006, Di 2011, Chen 2015) reporting large effects (SMD > 2.0). One study (Aydilek 2022) showed a negative effect (SMD = −1.70), possibly due to variations in intervention intensity or patient characteristics.

### Activities of daily living

**Figure 5:**
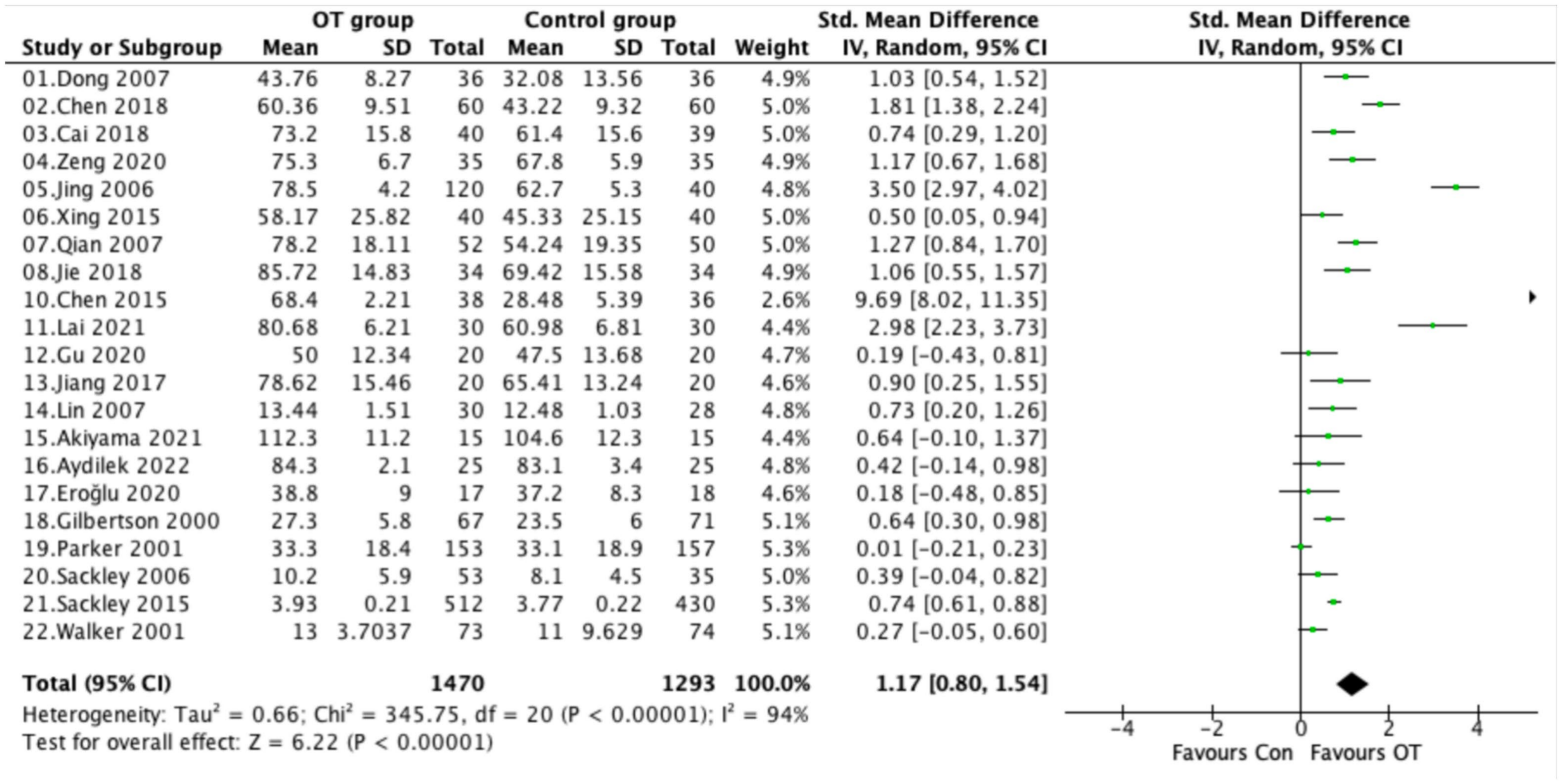
Forest plot comparing activities of daily living between the OT and control group.

Twenty-one studies evaluated the effect of occupational therapy on activities of daily living in stroke survivors. Due to substantial heterogeneity (*I*^2^ = 94%, *p* < 0.00001), a random-effects model was applied. The meta-analysis demonstrated a significant improvement in ADL outcomes among patients receiving occupational therapy compared with controls (SMD = 1.17, 95% CI [0.80, 1.54], *p* < 0.00001; Figure 5). Most studies reported effect sizes in favor of the OT group, ranging from small to very large. Notably, Chen 2015, Jing 2006, and Lai 2021 showed particularly strong effects (SMD > 2.0), suggesting that intensive or structured OT programs may yield greater functional gains. In contrast, studies such as Parker 2001 and Gu 2020 showed minimal or nonsignificant effects.

### Depressive symptoms

**Figure 6:**
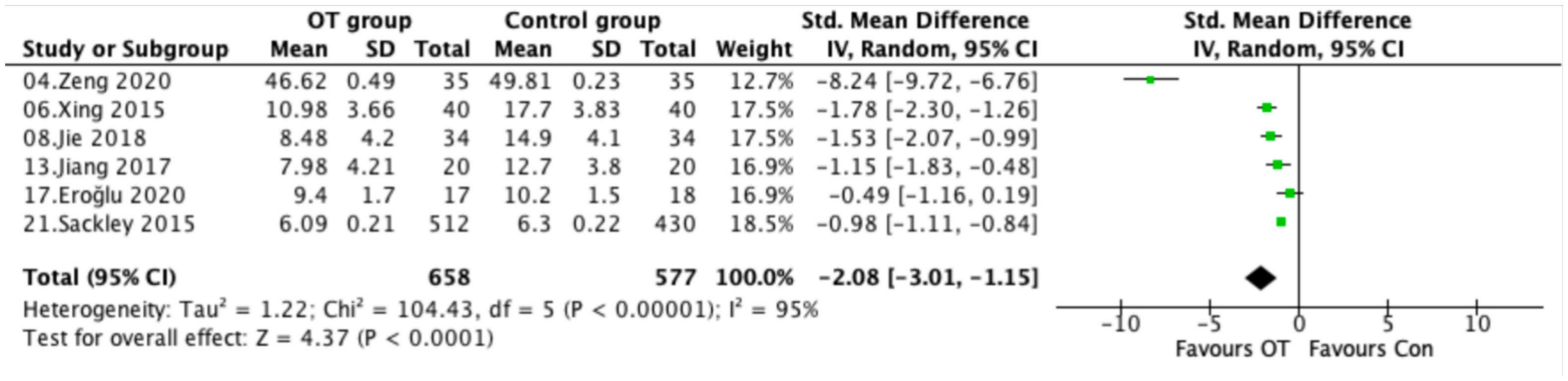
Forest plot comparing depressive symptoms between the OT and control group.

Six trials assessed depressive symptoms using validated instruments, including the Self-Rating Depression Scale (SDS), the 17-item Hamilton Depression Rating Scale (HAMD-17), the Hospital Anxiety and Depression Scale (HADS; depression component), and the 15-item Geriatric Depression Scale (GDS-15). Given substantial heterogeneity across studies (*I*^2^ = 95%, *p* < 0.00001), a random-effects model was applied. The pooled analysis showed that occupational therapy significantly reduced depressive symptoms compared with controls (SMD = −2.08, 95% CI −3.01 to −1.15, *p* < 0.00001; Figure 6).

### Subgroup analysis

#### By intervention setting

The effectiveness of OT varied significantly by intervention setting. For upper limb function, OT delivered in hospital or rehabilitation centers produced significant improvements compared to controls (SMD = 1.06, 95% CI 0.35–1.77; *I*^2^ = 95%). A markedly larger effect was observed in a home-based study (SMD = 5.72, 95% CI 4.67–6.77), though this result should be interpreted cautiously due to limited data. Subgroup differences were statistically significant (*p* < 0.00001). For ADL, OT yielded consistent benefits across all settings, with the greatest effects in home or community environments (SMD = 2.02, 95% CI 0.80–3.25), followed by hospital-based (SMD = 1.14, 95% CI 0.69–1.59) and residential care (SMD = 0.63, 95% CI 0.30–0.96); subgroup difference was significant (*p* = 0.03). Regarding depressive symptoms, significant improvements were noted in both hospital-based (SMD = −2.46, 95% CI −3.89 to −1.03) and residential settings (SMD = −0.98, 95% CI −1.11 to −0.84), suggesting structured clinical environments may offer psychological advantages. These findings highlight that setting-specific factors may modulate OT effectiveness, particularly in motor and psychological domains (Appendix Figures 4.1–4.3).

#### By OT intervention type

The type of OT intervention influenced outcomes across domains. For upper limb function, the greatest benefit was observed in OT combined with standard rehabilitation (SR) (SMD = 2.46, 95% CI 1.02–3.90). OT alone also showed a strong but imprecise effect (SMD = 3.21, 95% CI −1.65 to 8.07), likely due to small sample size. OT combined with PT showed a modest, non-significant effect (SMD = 0.53, 95% CI −0.37 to 1.42), and Screw Block® training did not demonstrate additional benefit (SMD = 0.32, 95% CI −0.40 to 1.04). For ADL, the strongest effect was observed with OT alone (SMD = 1.52, 95% CI 0.73–2.31), followed by OT + SR (SMD = 1.27) and OT + PT (SMD = 0.97). Again, OT + Screw Block® showed limited impact. In terms of depressive symptoms, all modalities produced favorable results: OT alone (SMD = −1.38, 95% CI −1.80 to −0.96), OT + SR (SMD = −0.98), and notably OT + PT (SMD = −3.40, 95% CI −6.34 to −0.45), although the latter was based on only three heterogeneous studies. Overall, OT combined with standard rehabilitation consistently demonstrated robust outcomes across domains, while OT alone showed substantial efficacy, especially in functional and psychological domains. Evidence supporting OT + PT or innovative methods remains limited and inconsistent (Appendix Figures 4.4–4.6).

#### By intervention duration

Intervention duration significantly impacted outcomes, particularly for motor recovery. For upper limb function, long-term interventions (>8 weeks) yielded the greatest improvements (SMD = 5.72, 95% CI 4.67–6.77), followed by short-term (≤4 weeks) (SMD = 1.60, 95% CI 0.43–2.77). Medium-term (5–8 weeks) interventions showed no significant effect (SMD = 0.50, 95% CI −0.61 to 1.61). Subgroup differences were statistically significant (*p* < 0.00001). For ADL, all durations produced clinically relevant benefits-long-term (SMD = 1.43), short-term (SMD = 1.24), and medium-term (SMD = 1.02)--with no significant subgroup differences (*p* = 0.79), suggesting duration-independent functional gains. Regarding depressive symptoms, both short-term (SMD = −1.50, 95% CI −2.11 to −0.90) and long-term (SMD = −0.98, 95% CI −1.11 to −0.84) interventions were effective, whereas the medium-term subgroup showed non-significant effects (SMD = −4.34, 95% CI −11.93 to 3.26), possibly due to limited data and heterogeneity (*p* = 0.17). Collectively, these results indicate that longer intervention durations may enhance outcomes in upper limb recovery and mood regulation, whereas ADL improvements appear consistent regardless of duration (Appendix Figures 4.7–4.9).

### Sensitivity analysis, publication bias, and evidence certainty

Sensitivity analyses confirmed the robustness of the findings across all three outcomes. Systematically removing each study did not substantially alter effect sizes or the direction of results, indicating that no single study disproportionately influenced the conclusions. Despite moderate to high heterogeneity in some outcomes, no dominant source was identified, supporting the stability of the pooled estimates (Appendix 5). The certainty of evidence, evaluated using the GRADE approach, was rated as low for all three outcomes. This downgrading reflected two serious domains: risk of bias across included studies, and substantial inconsistency as indicated by very high heterogeneity (*I*^2^ = 94–96%). Although meta-regression analyses did not identify specific study-level covariates explaining this heterogeneity, the magnitude of between-study variance remained clinically important and precluded a higher certainty rating. Accordingly, the pooled estimates should be interpreted with caution, and the findings are best regarded as preliminary evidence requiring confirmation from future well-standardized trials (Appendix 7). Visual inspection of funnel plots showed no clear asymmetry, indicating a low likelihood of publication bias (Appendix 6).

### Meta-regression

To investigate potential sources of heterogeneity, meta-regression analyses were conducted across all three outcome domains, examining four study-level covariates: mean age, dose intensity (h/week), intervention setting (hospital vs. community), and follow-up duration (weeks). Results are presented in Appendix 8. No covariate reached statistical significance in any outcome domain. For upper limb function, ADL, and depressive symptoms respectively, mean age (*p* = 0.62, 0.41, 0.74), dose intensity (*p* = 0.18, 0.33, 0.49), intervention setting (*p* = 0.49, 0.36, 0.67), and follow-up duration (*p* = 0.72, 0.58, 0.54) were all non-significant. These findings indicate that the substantial heterogeneity observed across studies could not be explained by these study-level variables alone, and likely reflects the inherent clinical and methodological diversity among included trials, including variability in OT protocols, patient case-mix, and outcome measurement instruments.

## Discussion

This systematic review and meta-analysis included 22 randomized controlled trials encompassing 2,833 stroke patients and suggested that OT may improve upper limb motor function, ADL, and depressive symptoms compared with standard care or conventional rehabilitation. However, given the substantial heterogeneity observed across all three outcome domains (*I*^2^ = 94–96%), these findings should be interpreted with caution; the pooled estimates reflect the average effect across a clinically and methodologically diverse body of evidence rather than a precise, universally applicable treatment effect. These findings were consistent across diverse clinical settings, intervention types, and durations. Notably, OT delivered in hospital or home settings, implemented either as a standalone intervention or combined with standard rehabilitation, was associated with greater functional and psychological benefits. Longer intervention durations (>8 weeks) appeared to yield superior improvements in motor recovery and mood, while gains in ADL were observed regardless of duration. Sensitivity analyses supported the robustness of these findings, and no substantial publication bias was detected. Overall, the evidence tentatively suggests that OT is an effective and adaptable rehabilitation strategy for enhancing both physical and emotional recovery in stroke survivors, though this conclusion must be tempered by the methodological limitations discussed below.

Occupational therapy is fundamentally grounded in a goal-oriented and client-centered approach, translating rehabilitation goals into meaningful, real-world task practice and participation. Goal-oriented therapy emphasizes training that is explicitly linked to functional objectives and task performance, thereby increasing practice intensity and supporting recovery of autonomy after stroke. In line with this principle, goal-oriented rehabilitation programs have been shown to improve functional autonomy (e.g., Barthel Index) alongside balance and gait-related outcomes in subacute stroke, highlighting that rehabilitation targets extend beyond upper-limb recovery alone. These concepts reinforce the rationale for OT as a cornerstone of post-stroke rehabilitation across multiple domains—including upper-limb function, walking-related mobility, and activities of daily living—by anchoring therapy in functional goals and task-specific training (34).

Notably, OT was associated with potentially meaningful improvements in upper limb function, a critical determinant of post-stroke independence due to the central role of the upper limbs in daily activities (35). It is worth noting, however, that the pooled SMD of 1.42 for upper limb function, while statistically large by conventional thresholds (SMD > 0.8), must be interpreted in the context of clinical meaningfulness. For the Fugl-Meyer Assessment–Upper Extremity (FMA-UE), the minimal clinically important difference (MCID) has been estimated at approximately 4.25–7.25 points in subacute stroke populations. Whether the observed pooled effect translates to a change of this magnitude in absolute scale terms will vary across studies depending on baseline severity and the specific instruments used, and cannot be uniformly confirmed from the standardized effect size alone. Clinicians should therefore exercise caution when extrapolating these statistical findings to individual patient care. These findings are consistent with previous research (36–38), and it has been hypothesized that the observed benefits may be attributed to the neuroplastic potential of the brain. Based on evidence from external neuroimaging and neurophysiological studies—rather than direct measurement within the included RCTs, none of which assessed neuroplasticity outcomes—it is theorized that by engaging patients in structured, task-oriented activities, OT may stimulate axonal and dendritic growth, promoting reorganization of damaged neural pathways and facilitating motor recovery (39, 40). These mechanistic explanations should therefore be regarded as plausible hypotheses informed by the broader neuroscience literature, rather than conclusions directly supported by the present data. Additionally, active involvement in OT promotes limb mobilization and muscle activation, helping to correct abnormal motor patterns and reinforce neuromuscular control (41).

Beyond motor function, OT appeared to enhance performance in ADL, aligning with earlier findings (9, 10, 42). Several studies reported notably large effect sizes in this domain; in particular, Chen 2015 (SMD = 9.69) and Zeng 2020 should be regarded as statistical outliers, as their effect sizes are implausibly large by conventional standards (SMD > 0.8 is already considered “large” in meta-analytic literature). Upon re-verification, the data extracted from Chen 2015 were confirmed to be consistent with the original publication; the extreme SMD appears to reflect the unusually small standard deviations reported in that study rather than a data extraction error. Importantly, the sensitivity analysis reported in Appendix 5, in which each study was systematically removed in turn, demonstrated that excluding Chen 2015 and Zeng 2020 did not substantially alter the overall direction or magnitude of the pooled ADL effect, confirming the robustness of the primary finding despite the presence of these outliers. Nonetheless, these studies should be interpreted with caution and their disproportionate influence on the pooled estimate acknowledged. This effect likely stems from the functional nature of OT interventions, which integrate real-life tasks such as grooming, feeding, and dressing, alongside exercises targeting strength, coordination, and fine motor skills (43). These activities are designed to mirror everyday demands, follow a progression from gross to fine movements, and incorporate elements that maintain patient engagement—features that contribute to sustained participation and meaningful functional gains. Furthermore, OT’s positive impact on ADL may translate into improved quality of life, as supported by earlier studies (44–47). By restoring physical independence, OT may also enhance psychological wellbeing and cognitive function (48), particularly when interventions include creative or leisure-based tasks like knitting, painting, and sculpting, which can enhance motivation and emotional resilience.

The analysis also suggested that OT may effectively reduce depressive symptoms in stroke patients, reinforcing findings from Huang Hongyan and colleagues on the utility of behavioral therapies in post-stroke depression (49, 50). Depressive symptoms are common following stroke and are often linked to loss of function and autonomy. The degree of neurological impairment has been shown to correlate with depressive severity (51); therefore, improvements in physical capabilities via OT may also foster emotional recovery. Success in completing meaningful tasks may provide a sense of accomplishment, reinforcing positive affect and reducing psychological distress. However, not all studies observed this benefit. For example, Walker et al. (52) reported no significant mood improvement post-OT, potentially due to delayed intervention or differences in therapeutic approach. These inconsistencies underscore the need for early, individualized OT as part of comprehensive stroke rehabilitation. Clinicians are encouraged to inform patients and caregivers about the multifaceted benefits of OT and to promote active, personalized participation as a means of enhancing functional recovery, psychological health, and overall wellbeing.

Subgroup analyses revealed that the effectiveness of OT varies considerably depending on intervention setting, delivery mode, and duration. Home- and hospital-based interventions demonstrated superior outcomes compared to residential settings, particularly for upper limb and psychological recovery. These differences likely reflect variations in therapeutic intensity, patient engagement, and resource availability across environments, underscoring the need to tailor OT programs to context-specific factors. Intervention type also shaped outcomes. OT combined with standard rehabilitation consistently produced positive effects across domains, suggesting synergy between multidisciplinary approaches. Interestingly, OT alone yielded comparable or even greater benefits in ADL and mood outcomes, possibly due to focused task-oriented training. In contrast, combining OT with physical therapy or device-based strategies (e.g., Screw Block®) showed inconsistent results, highlighting that therapeutic specificity may outweigh intervention complexity. Intervention duration further influenced efficacy. Long-term OT was most effective for motor recovery and depressive symptoms, supporting the role of sustained engagement in neuroplastic and emotional adaptation. In contrast, ADL improvements appeared less duration-dependent, suggesting that early functional gains may be achievable with shorter interventions. Together, these findings emphasize that OT is not a one-size-fits-all intervention; rather, its impact is shaped by how, where, and for how long it is delivered. Future research should focus on optimizing intervention strategies for different patient profiles and clinical settings.

This review extends the existing evidence base on OT after stroke in several important ways. First, recent syntheses have either focused on chronic stroke populations and predominantly evaluated ADL outcomes, with limited or non-significant effects reported for broader physical domains, or have addressed adjunctive modalities (e.g., FES/tDCS) added to OT rather than OT itself (53, 54). In contrast, the present meta-analysis provides an updated evaluation of OT as a standalone intervention, with a primary emphasis on upper-limb function, and additionally synthesizes effects on ADL and depressive symptoms, thereby capturing both physical and psychological domains of post-stroke rehabilitation. Moreover, by including RCTs published in both English and Chinese and conducting prespecified subgroup analyses by setting, OT type, and duration, we offer clinically actionable insights into potential sources of heterogeneity and conditions under which OT may confer greater benefit.

Several limitations of this review should be acknowledged. First, substantial heterogeneity was observed across outcomes, which, despite the use of random-effects models, subgroup analyses, and meta-regression, may limit the generalizability of the findings. Second, many included trials provided insufficient detail regarding allocation concealment and blinding, raising concerns about potential risk of bias. Third, considerable variability in OT protocols, outcome measures, and follow-up durations limited direct comparability across studies. Importantly, although upper limb motor function was a primary outcome, spasticity—an important factor that can substantially impair upper limb use after stroke—was not consistently reported or quantitatively analyzed across the included trials, precluding a separate synthesis of its effects. In addition, cognitive impairment and language disorders (e.g., aphasia), which are highly prevalent after stroke and critically influence functional independence and rehabilitation participation, were not systematically assessed in this review due to insufficient and heterogeneous reporting. Furthermore, as the literature search was restricted to English and Chinese publications, trials conducted in other languages—including potentially significant European and Japanese studies—may have been missed, representing a language bias that could limit the generalizability of our findings to non-Asian clinical contexts. These unmeasured domains may partly account for residual heterogeneity and should be addressed in future trials. Finally, depressive symptoms were reported in only a limited number of studies, warranting cautious interpretation. Future high-quality, multicenter RCTs incorporating standardized assessments of motor impairment, spasticity, cognitive and language function, as well as long-term follow-up, are needed to provide a more comprehensive evaluation of the effects of occupational therapy after stroke. Future studies should also examine the potential influence of environmental and socioeconomic conditions on OT outcomes, as these factors—though not measured in the trials included in the present review—may substantially moderate the effectiveness of rehabilitation interventions across different healthcare systems and patient populations.

## Conclusion

This meta-analysis suggests that OT offers significant benefits for stroke patients, particularly in enhancing upper limb function, improving activities of daily living, and alleviating depressive symptoms. These effects were generally consistent across different intervention settings, types, and durations, though longer-term and structured OT programs may yield greater improvements in motor and psychological outcomes. While the overall findings support the integration of OT into post-stroke rehabilitation, the presence of methodological limitations and heterogeneity among included studies underscores the need for cautious interpretation. Future high-quality, standardized, and multicenter randomized controlled trials are warranted to strengthen the evidence base and inform clinical practice more reliably.

## Data Availability

The original contributions presented in the study are included in the article/Supplementary material, further inquiries can be directed to the corresponding author.
