# Supplementary material for: Effect of occupational therapy on upper limb function and related rehabilitation outcomes after stroke: a systematic review and meta-analysis
Source: Front Neurol. 2026 Apr 23;17:1692088. doi: 10.3389/fneur.2026.1692088 (PMC13149191; doi:10.3389/fneur.2026.1692088)
Supplement: Supplementary file 1 [file Supplementary_file_1.docx]

**Appendix 1: PRISMA Checklist.**

| **Section and Topic** | **Item #** | **Checklist item** | **Location where item is reported** |
| --- | --- | --- | --- |
| **TITLE** | | |  |
| Title | 1 | Identify the report as a systematic review. | P1 |
| **ABSTRACT** | | |  |
| Abstract | 2 | See the PRISMA 2020 for Abstracts checklist. | P1 |
| **INTRODUCTION** | | |  |
| Rationale | 3 | Describe the rationale for the review in the context of existing knowledge. | P2 |
| Objectives | 4 | Provide an explicit statement of the objective(s) or question(s) the review addresses. | P2 |
| **METHODS** | | |  |
| Eligibility criteria | 5 | Specify the inclusion and exclusion criteria for the review and how studies were grouped for the syntheses. | P3 |
| Information sources | 6 | Specify all databases, registers, websites, organisations, reference lists and other sources searched or consulted to identify studies. Specify the date when each source was last searched or consulted. | P2 |
| Search strategy | 7 | Present the full search strategies for all databases, registers and websites, including any filters and limits used. | P2/appendix2 |
| Selection process | 8 | Specify the methods used to decide whether a study met the inclusion criteria of the review, including how many reviewers screened each record and each report retrieved, whether they worked independently, and if applicable, details of automation tools used in the process. | P3 |
| Data collection process | 9 | Specify the methods used to collect data from reports, including how many reviewers collected data from each report, whether they worked independently, any processes for obtaining or confirming data from study investigators, and if applicable, details of automation tools used in the process. | P3 |
| Data items | 10a | List and define all outcomes for which data were sought. Specify whether all results that were compatible with each outcome domain in each study were sought (e.g. for all measures, time points, analyses), and if not, the methods used to decide which results to collect. | P3 |
|  | 10b | List and define all other variables for which data were sought (e.g. participant and intervention characteristics, funding sources). Describe any assumptions made about any missing or unclear information. | P3 |
| Study risk of bias assessment | 11 | Specify the methods used to assess risk of bias in the included studies, including details of the tool(s) used, how many reviewers assessed each study and whether they worked independently, and if applicable, details of automation tools used in the process. | P3 |
| Effect measures | 12 | Specify for each outcome the effect measure(s) (e.g. risk ratio, mean difference) used in the synthesis or presentation of results. | P3 |
| Synthesis methods | 13a | Describe the processes used to decide which studies were eligible for each synthesis (e.g. tabulating the study intervention characteristics and comparing against the planned groups for each synthesis (item #5)). | P3 |
|  | 13b | Describe any methods required to prepare the data for presentation or synthesis, such as handling of missing summary statistics, or data conversions. | P3 |
|  | 13c | Describe any methods used to tabulate or visually display results of individual studies and syntheses. |  |
|  | 13d | Describe any methods used to synthesize results and provide a rationale for the choice(s). If meta-analysis was performed, describe the model(s), method(s) to identify the presence and extent of statistical heterogeneity, and software package(s) used. | P3 |
|  | 13e | Describe any methods used to explore possible causes of heterogeneity among study results (e.g. subgroup analysis, meta-regression). | P3 |
|  | 13f | Describe any sensitivity analyses conducted to assess robustness of the synthesized results. | P3 |
| Reporting bias assessment | 14 | Describe any methods used to assess risk of bias due to missing results in a synthesis (arising from reporting biases). | P3 |
| Certainty assessment | 15 | Describe any methods used to assess certainty (or confidence) in the body of evidence for an outcome. | P3 |
| **RESULTS** | | |  |
| Study selection | 16a | Describe the results of the search and selection process, from the number of records identified in the search to the number of studies included in the review, ideally using a flow diagram. | P4 |
|  | 16b | Cite studies that might appear to meet the inclusion criteria, but which were excluded, and explain why they were excluded. | P5 |
| Study characteristics | 17 | Cite each included study and present its characteristics. | P5 |
| Risk of bias in studies | 18 | Present assessments of risk of bias for each included study. | P6 |
| Results of individual studies | 19 | For all outcomes, present, for each study: (a) summary statistics for each group (where appropriate) and (b) an effect estimate and its precision (e.g. confidence/credible interval), ideally using structured tables or plots. | P6 |
| Results of syntheses | 20a | For each synthesis, briefly summarise the characteristics and risk of bias among contributing studies. | P7 |
|  | 20b | Present results of all statistical syntheses conducted. If meta-analysis was done, present for each the summary estimate and its precision (e.g. confidence/credible interval) and measures of statistical heterogeneity. If comparing groups, describe the direction of the effect. | P7 |
|  | 20c | Present results of all investigations of possible causes of heterogeneity among study results. | P8 |
|  | 20d | Present results of all sensitivity analyses conducted to assess the robustness of the synthesized results. | appendix |
| Reporting biases | 21 | Present assessments of risk of bias due to missing results (arising from reporting biases) for each synthesis assessed. | P5 |
| Certainty of evidence | 22 | Present assessments of certainty (or confidence) in the body of evidence for each outcome assessed. | P9 |
| **DISCUSSION** | | |  |
| Discussion | 23a | Provide a general interpretation of the results in the context of other evidence. | P9 |
|  | 23b | Discuss any limitations of the evidence included in the review. | P11 |
|  | 23c | Discuss any limitations of the review processes used. | P11 |
|  | 23d | Discuss implications of the results for practice, policy, and future research. | P10 |
| **OTHER INFORMATION** | | |  |
| Registration and protocol | 24a | Provide registration information for the review, including register name and registration number, or state that the review was not registered. | P2 |
|  | 24b | Indicate where the review protocol can be accessed, or state that a protocol was not prepared. |  |
|  | 24c | Describe and explain any amendments to information provided at registration or in the protocol. |  |
| Support | 25 | Describe sources of financial or non-financial support for the review, and the role of the funders or sponsors in the review. | P11 |
| Competing interests | 26 | Declare any competing interests of review authors. | P11 |
| Availability of data, code and other materials | 27 | Report which of the following are publicly available and where they can be found: template data collection forms; data extracted from included studies; data used for all analyses; analytic code; any other materials used in the review. | P11 |

**Appendix 2: Search strategy**

Search strategy of **PubMed**

| **#** | **Searches** |
| --- | --- |
| 1 | Stroke[MeSH Terms] OR Strokes[Title/Abstract] OR Cerebrovascular Accident[Title/Abstract] OR Cerebrovascular Accidents[Title/Abstract] OR Cerebral Stroke[Title/Abstract] OR Cerebral Strokes[Title/Abstract] OR Stroke, Cerebral[Title/Abstract] OR Strokes, Cerebral[Title/Abstract] OR Cerebrovascular Apoplexy[Title/Abstract] OR Apoplexy, Cerebrovascular[Title/Abstract] OR Vascular Accident, Brain[Title/Abstract] OR Brain Vascular Accident[Title/Abstract] OR Brain Vascular Accidents[Title/Abstract] OR Vascular Accidents, Brain[Title/Abstract] OR Cerebrovascular Stroke[Title/Abstract] OR Cerebrovascular Strokes[Title/Abstract] OR Stroke, Cerebrovascular[Title/Abstract] OR Strokes, Cerebrovascular[Title/Abstract] OR Apoplexy[Title/Abstract] OR CVA[Title/Abstract] OR CVAs[Title/Abstract] OR Stroke, Acute[Title/Abstract] OR Acute Stroke[Title/Abstract] OR Acute Strokes[Title/Abstract] OR Strokes, Acute[Title/Abstract] OR Cerebrovascular Accident, Acute[Title/Abstract] OR Acute Cerebrovascular Accident[Title/Abstract] OR Acute Cerebrovascular Accidents[Title/Abstract] OR Cerebrovascular Accidents, Acute[Title/Abstract] |
| 2 | Occupational Therapy[MeSH Terms] OR Occupational Therapies[Title/Abstract] OR Therapy, Occupational[Title/Abstract] OR Therapies, Occupational[Title/Abstract] OR Occupational Therapy Intervention[Title/Abstract] OR Occupational Therapy Interventions[Title/Abstract] OR Intervention, Occupational Therapy[Title/Abstract] OR Interventions, Occupational Therapy[Title/Abstract] OR OT Intervention[Title/Abstract] OR OT Interventions[Title/Abstract] OR Occupational Therapist[Title/Abstract] OR Occupational Therapists[Title/Abstract] |
| 3 | ((((Randomized Controlled Trials as Topic[MeSH Terms]) OR (Clinical Trials, Randomized[Title/Abstract])) OR (Trials, Randomized Clinical[Title/Abstract])) OR (Controlled Clinical Trials, Randomized[Title/Abstract])) OR (RCT[Title/Abstract]) |
| 4 | #1 AND #2 AND #3 |

**Appendix 3. Detailed Description of Intervention and Control Conditions**

| Study | Treatment group | Control group | Outcome measures |
| --- | --- | --- | --- |
| 01.Dong 2007 | OT + PT (simultaneous) for 6 weeks:  OT: daily (60 min/session), focused on ADL re-training (e.g., dressing, eating, transferring), task-oriented therapy, fine motor training of affected upper limb, use of assistive devices, and compensatory techniques.  One-on-one sessions; additional ADL practice guided by nurses and caregivers outside therapy hours. | PT: daily (60 min/session), including positioning, passive joint mobilization, bridging, PNF, sitting/standing balance, gait training. | FMA for motor function of the paretic limb;  MBI for activities of daily living (ADL), categorized into five functional levels;  Assessments performed at baseline, week 2, week 4, and week 6 by a multidisciplinary team (physician, nurse, therapist);  Adverse events and complications monitored throughout. |
| 02.Chen 2018 | OT + motor therapy (4 weeks): individualized task-based OT (20–30 min, twice daily, 6 days/week × 3 cycles), including proximal-to-distal upper limb control, joint coordination with assistive devices, fine motor training (e.g., ring stacking, screw turning), ADL training (e.g., washing, eating, dressing). One-on-one sessions; family-assisted practice encouraged post-discharge. | Motor therapy only: 45 min/session, 5 times/week × 4 weeks, including positioning, spasticity reduction, ROM, balance, gait, and transfer training based on stroke stage and individual needs. | Fugl-Meyer Assessment (FMA, max 66) for motor function; Wolf Motor Function Test (WMFT, max 75) for upper limb motor performance; Barthel Index (BI, max 100) for ADL. Evaluated at baseline and at 3 months post-intervention. |
| 03.Cai 2018 | Early OT initiated after 14 days of motor therapy; 60 min/day, once daily. OT focused on fine motor training of the affected limb, compensatory strategies with unaffected side, and ADL re-training (e.g., eating, dressing, grooming, transferring). Based on Brunnstrom and Bobath principles. One-on-one therapist-led sessions; caregiver and nurse-assisted ADL training outside therapy. Psychological support provided to enhance engagement. | Standard motor therapy only: progressive training based on recovery stage, including passive/active limb movement, rolling, bridging, sitting, standing, balance, walking, and stair practice. | Fugl-Meyer Assessment (FMA) for upper and lower limb motor function; Modified Barthel Index (MBI) for ADL. Assessed post-intervention by the same trained team. |
| 04.Zeng 2020 | OT + PT (6 weeks): PT included positioning and passive joint movement during bed rest. OT focused on re-training of ADLs (e.g., turning, dressing, eating, transferring) to improve self-care ability. Additional components included psychological support, pre-intervention planning, caregiver education, and supervised OT safety management. | PT only: positioning and passive joint movement during bed rest; no OT intervention or psychological support provided. | Self-rating Anxiety Scale (SAS) and Self-rating Depression Scale (SDS) for emotional status; ADL ability scored before discharge by physician, nurse, and family. |
| 05.Jing 2006 | OT + PT: individualized PT (40–50 min/day, avg. 7 weeks), including positioning, passive joint movement, bridging, PNF, sitting/standing balance, and gait training. Concurrent early OT (45–60 min/day): re-training of ADLs (e.g., turning, dressing, eating, transferring), task-specific therapy, fine motor training for the affected upper limb, use of assistive/self-help devices, and compensatory strategies. One-on-one sessions; caregivers encouraged to minimize assistance. | PT only: same PT protocol as treatment group (40–50 min/day, avg. 7 weeks), provided by a therapist one-on-one. No OT intervention. | Fugl-Meyer Assessment (FMA): 100-point total (upper limb: 66, lower limb: 34), with <50 indicating severe motor impairment. Barthel Index (BI): 10 items, 0–100 scale, <60 indicating dependency. Baseline assessment within 24 h of rehab start, followed by evaluations every 2 weeks by the same therapist. |
| 06.Xing 2015 | OT + PT: PT (40–50 min/day, avg. 7 weeks), including positioning, passive joint movement, bridging, PNF, balance, and gait training, all delivered one-on-one. Concurrent early OT (45–60 min/day), focused on re-training ADLs (e.g., turning, dressing, eating, transferring), task-specific upper limb therapy, fine motor training, use of assistive/self-help devices, and compensatory strategies. Family/nurse supervision encouraged outside therapy to reduce dependency. | PT only: same protocol as treatment group (40–50 min/day, avg. 7 weeks), delivered one-on-one. No OT provided. | Fugl-Meyer Assessment (FMA), total 100 points (upper limb: 66; lower limb: 34); <50 = severe impairment. Barthel Index (BI), total 100 points; <60 = dependent. Assessed at baseline (within 24h of rehab start) and every 2 weeks by the same therapist. |
| 07.Qian 2007 | OT only: initiated after vital signs stabilized. Sessions twice daily, 45 min each, 5 days/week. Early-stage OT included passive ROM (shoulder, wrist), scapular mobilization, position adaptation, and assisted transitions. Recovery-phase OT included task-specific training (e.g., sanding board, rollers, pegboard, weaving, clay work, drawing, folding, grasping), combined with use of orthoses, assistive, and self-help devices. Activities were individualized based on patient needs. | No rehabilitation provided. Patients instructed to ambulate independently once stable; no structured therapy. | Brunnstrom stages (I–VI) for upper limb and hand motor function; Barthel Index (BI) for ADL. Each patient assessed at baseline (after stabilization), and at 1 and 3 months post-treatment by trained evaluators. |
| 08.Jie 2018 | OT only: initiated after stabilization. Training included passive ROM for shoulder/wrist, scapular mobilization, postural adaptation, assisted limb flexion, and upper limb loading. Early-stage OT emphasized grip training (e.g., using glass balls, rubber rings), reaching tasks (e.g., head, mouth, grooming). Recovery-phase included task-oriented activities (e.g., sanding, rollers, pegboard, drawing, clay, origami, stacking cones, picking blocks, towel twisting, bottle opening). Delivered twice daily, 45 min/session, 5 days/week. | Routine medical treatment and general rehabilitation only; no specific OT intervention reported. | Hamilton Depression Scale (HAMD) for emotional status; Barthel Index (BI/MBI) for ADL ability. Outcomes compared between groups post-intervention. |
| 09.Di 2011 | OT + PT: both groups received standard stroke treatment and motor therapy after stabilization. PT included passive/active movement, postural training, bridging, balance, gait training, and motor imagery (10–12 min/day, with guided scripts). OT added sequential, task-oriented training starting in supine/sitting, progressing from proximal to distal joints, emphasizing posture, coordination, endurance, and hand-eye tasks (e.g., sanding board, rollers, pegboard, ball/pinching tasks, fine motor games like assembly and mosaic tasks). | PT only: passive and active joint movement, postural transitions, bridging, gait, balance training, and motor imagery. No occupational therapy applied. | Fugl-Meyer Assessment (FMA) for upper limb motor function, 33 items (max 66 points). Evaluated at baseline and at weeks 2, 4, 6, and 8. Higher scores indicate better recovery. |
| 10.Chen 2015 | Home-based OT + standard secondary stroke prevention. OT prescribed via outpatient follow-up every 2 weeks for 3 months. Task: wiping exercises tailored to upper limb function level, from passive-assisted wiping (with unaffected limb) to active wiping on varied surfaces and angles using resistance (e.g., textured boards). Activities incorporated daily living tasks (e.g., face washing, brushing teeth, combing, eating). Self-training performed 3×/day, 40 min/session, ≥5 days/week, supervised by family. | Standard secondary stroke prevention only (per 2014 AHA/ASA guidelines); no home-based OT assigned. Routine outpatient follow-up every 2 weeks. | Fugl-Meyer Assessment (FMA) for upper limb motor function; Barthel Index (BI) for ADL. Both measured at discharge and 3 months post-discharge. Higher scores indicate better function. |
| 11.Lai 2021 | OT + standard rehab: patients received structured “occupation-based life reconstruction” OT in both therapy and ward settings. OT: 45 min/day, 5 days/week, 4 weeks. Bedside extension training included functional ADLs (e.g., turning, transferring, eating, grooming, toileting), with active involvement of the affected limb. Tasks included assistive use of the affected hand during eating, washing, combing, shaving, etc. Rehabilitation nurses coordinated with OT therapists to supervise, document, and adjust ward-based training. | Standard rehabilitation only: conventional drug therapy and general rehab including physical therapy, physical modalities, and acupuncture. No OT extension training provided. | Fugl-Meyer Assessment (FMA, max 66) for upper limb motor function; Modified Barthel Index (MBI, max 100) for ADL. Both assessed pre-treatment and after 4 weeks. |
| 12.Gu 2020 | OT + basic rehab: patients received PT and physical therapy plus OT (45 min/day, 5 days/week). OT included neuromuscular facilitation (Bobath techniques), upper limb ROM training using hand splints and assisted movements, fine motor training (e.g., screwing, beading, pegboard), hand-eye coordination, and individualized or group-based ADL training based on patient needs and interests. | Basic rehab only: PT and physical therapy without any occupational therapy. | Modified Barthel Index (MBI, max 100) for ADL function, assessed pre-treatment and at 6 weeks post-treatment. |
| 13.Jiang 2017 | Game-based OT + standard rehab: both groups received early rehab and oral Deanxit for 2 weeks. Treatment group additionally received interest-oriented OT (1 h/session, 2×/day, 5 days/week × 2 weeks), including: (1) music-assisted upper limb exercises; (2) modified OT games (e.g., pegboards with letters/numbers, rolling smiley cylinders, sanding on patterned boards, bell games); (3) ADL group games (e.g., face-wiping relay, musical chairs); and (4) music and reading clubs for cognitive and emotional engagement. Activities conducted in small interactive groups. | Early conventional physical rehab only + oral Deanxit (same dose and duration); no OT. | Hamilton Depression Scale (HAMD-17) for depressive symptoms (range 0–52); Modified Barthel Index (MBI, max 100) for ADL. Both assessed before and after the 2-week intervention by the same therapist. |
| 14.Lin 2007 | OT + PT: all patients received stroke-specific drug treatment. PT (40 min, 2×/day, 5 days/week × 4 weeks) included positioning, ROM, anti-spasticity techniques, balance, gait, and transfers (Bobath & Rood-based). OT (40 min/day) was individualized and stage-specific: (1) Flaccid phase – strength, pain relief, shoulder stability (e.g., bilateral arm movements); (2) Spastic phase – tone and ROM training (e.g., cylindrical rolling, sanding, finger separation); (3) Recovery phase – coordination, endurance, fine motor training (e.g., pegboard, modeling clay, blocks, threading, games); plus group-based ADL exercises (e.g., music-assisted movements, ball/hoop games). | PT only: same PT program as treatment group; no OT intervention. | Brunnstrom stages for upper limb motor function; Functional Comprehensive Assessment (FCA) upper limb sub-items (eating, grooming, dressing; total score = 18). Assessments at baseline, 1 month, and 3 months post-treatment by blinded evaluators. |
| 15.Akiyama 2021 | Screw Block® + conventional OT: both groups received OT 5 days/week for 3 weeks. EG group received 20–40 min/day of conventional OT (e.g., pegboard, ADL task practice) plus 20 min/day of Screw Block® training, involving bimanual manipulation and task progression based on complexity. Tasks were continued across sessions if not completed. Training was one-on-one; progression encouraged. | Conventional OT only: 40–60 min/day, 5 days/week for 3 weeks. Included upper limb training and ADL tasks, excluding Screw Block® activities. | Outcome measures included the Simple Test for Evaluating Hand Function (STEF) as the primary endpoint, assessing affected hand dexterity. Secondary outcomes comprised Ueda’s 12-grade hemiplegia scale (upper limb function), JASMID (paralyzed limb use in ADLs), General Self-Efficacy Scale (GSES), visual analogue scales (emotion-related states), and the Functional Independence Measure (FIM) evaluating both motor and cognitive independence. |
| 16.Aydilek 2022 | OT + standard rehab: all participants received conventional PT, including passive/active/active-assisted ROM, strength, transfer, gait, and stair training. The treatment group additionally received OT delivered by a trained occupational therapist using task-based tools (e.g., therapy putty, balls, colored cylinders, pegboards, blocks, screws, and a functional training board with handles, locks, faucets, lights). | Standard rehab only: PT including ROM training, strength, gait, transfers, and stair climbing. No OT intervention or assistive tool training provided. | Fugl-Meyer Assessment for upper limb motor function (FMA); Action Research Arm Test (ARAT); Barthel Index (BI) for ADL. All assessments conducted at baseline and week 6. |
| 17.Eroğlu 2020 | OT + standard rehab (SR): all patients received SR 5 days/week for 45 min/session, including passive/active ROM, stretching, strengthening, transfer, gait, stair climbing, and weight shifting under therapist supervision. Additionally, OT group received individualized OT 3 days/week (Mon, Wed, Fri) for 8 weeks. Each patient selected 5 functional tasks based on personal limitations, with weekly adjustments in task type or difficulty. | Standard rehab (SR) only: same frequency and duration as treatment group (5×/week, 45 min/session for 8 weeks), including general physical rehab activities. No OT intervention. | Grip strength, pinch strength (dynamometer); Purdue Pegboard Test; Nottingham Extended ADL (NEADL); PASS; SF-36; HADS (anxiety/depression); ADL performance and quality of life. |
| 18.Gilbertson 2000 | Individualized home-based OT + standard care: 6-week, patient-centered OT program (~10 sessions, 30–45 min each), developed via focus groups with patients, caregivers, and community OT providers. Goals targeted ADLs, home/leisure re-engagement. Therapists collaborated with patients on goal attainment and coordinated with external services for support and equipment. | Standard multidisciplinary rehab: inpatient rehab, selected pre-discharge home visits, stroke clinic follow-ups, and referrals to day hospitals. No structured home-based OT program provided. | Primary: Nottingham Extended ADL Scale (NEADL); composite outcome (Barthel-based) for deterioration or death. Secondary: Barthel Index, outpatient service satisfaction, healthcare resource use (e.g., readmission, staff time, equipment), and self-rated health status. Assessments at baseline, 8 weeks, and 6 months. |
| 19.Parker 2001 | Home-based OT (up to 6 months): minimum of 10 sessions (≥30 min each). Participants were assigned to one of two subgroups: (1) ADL-focused group – practiced independence in self-care tasks (e.g., meal prep, outdoor mobility); (2) Leisure-focused group – practiced leisure-related tasks and relevant ADL components. Therapists recorded session dates and durations using standardized forms. | No OT intervention during the trial. All participants continued to receive usual community rehabilitation services as available (e.g., day hospital visits). |  |
| 20.Sackley 2006 | Individualized OT (3 months): delivered by experienced therapists using a client-centered approach. Included initial 1-hour assessment to set ADL-based goals (e.g., feeding, dressing, toileting, mobility). Interventions involved task-specific practice, environmental modification (e.g., aids, adaptations), and therapeutic techniques (e.g., stretching, splinting). Also included staff/caregiver education on promoting independence and continuing therapy. | Usual care only: no occupational therapy provided. UK care homes did not routinely provide OT or ADL training. No designated staff responsible for functional rehabilitation. | Primary: Barthel Index (BI, 0–20) for self-care independence. Secondary: “poor global outcome” (BI deterioration or death), Rivermead Mobility Index (RMI, 0–15). Cognitive status assessed at baseline using Orientation-Memory-Concentration Test. Assessments at baseline, 3 months, and 6 months by blinded assessors. |
| 21.Sackley 2015 | Client-centered OT in care homes (3 months): individualized OT based on functional assessment and goal setting. Interventions targeted personal ADLs (e.g., dressing, feeding, toileting, mobility), using task-specific practice, environmental adaptations (e.g., bed levers, raised toilet seats), and therapist-led training. Education workshops provided to staff on stroke-related care, mobility, equipment use, and positioning. Frequency/duration based on resident’s goals and needs. | Usual care: no occupational therapy provided during trial. Care homes did not routinely offer OT or ADL-focused rehabilitation. Staff received training after 12-month follow-up. | Primary: Barthel Index (BI, 0–20), assessed at 3, 6, and 12 months. Secondary: Rivermead Mobility Index (RMI), Geriatric Depression Scale (GDS-15), EQ-5D-3L. Baseline cognitive screening via MMSE and Sheffield test. Outcomes assessed by blinded assessors. |
| 22.Walker 2001 | Up to 5 months of home-based occupational therapy (1–15 visits, avg. 6), starting within 1 month post-stroke. Focus: promote independence in personal and extended ADLs. Delivered by trained OT. | No OT intervention. Standard community care only. | Primary: Nottingham EADL Scale. Secondary: Barthel Index, GHQ-28 (patients & carers). Assessments at 6 and 12 months. Blinded assessors. |

**Appendix 4: Subgroup analysis**

**Figure 4.1. Subgroup analysis of upper limb function by intervention settings**

**
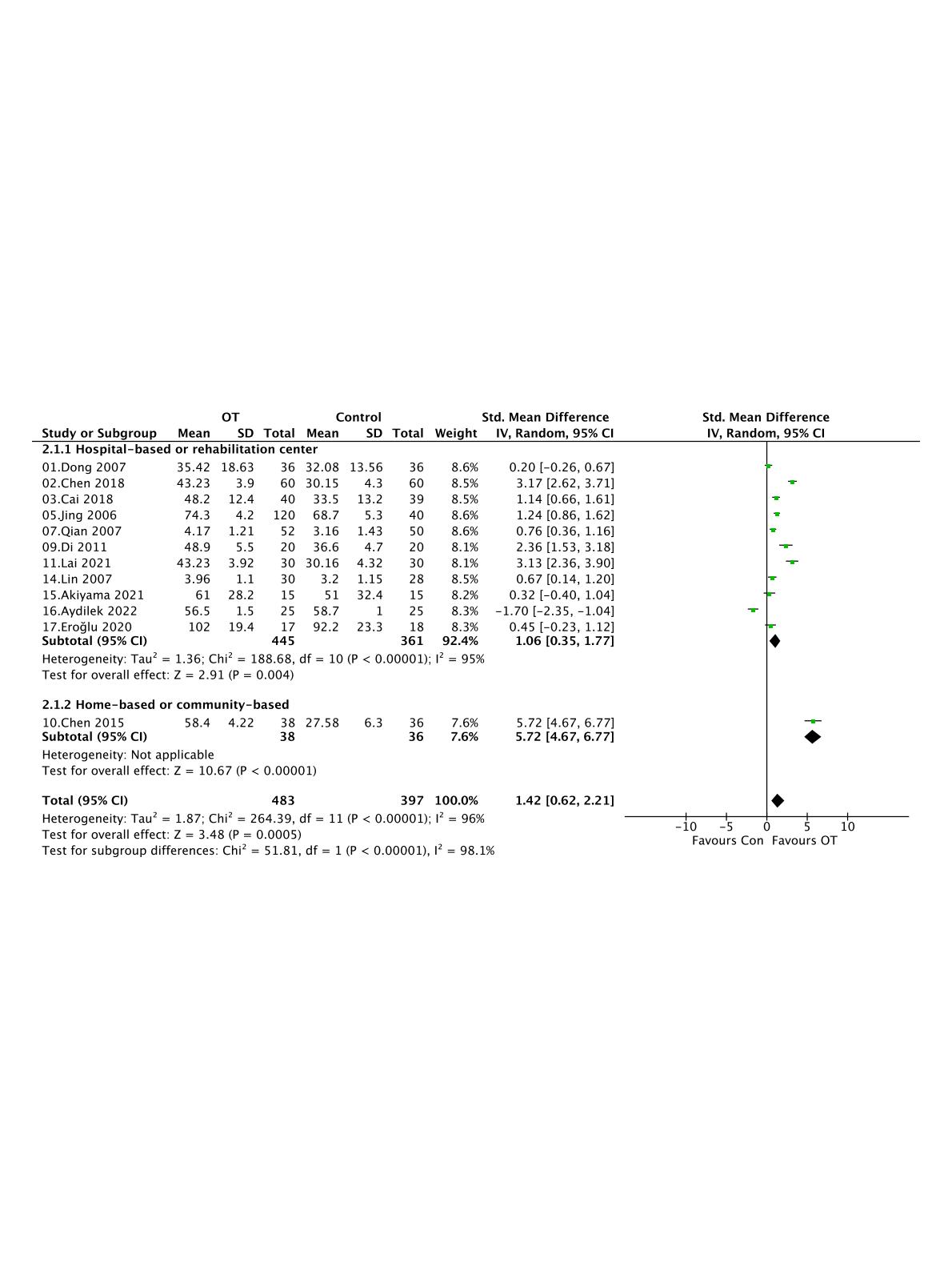
**

**Figure 4.2. Subgroup analysis of Activities of daily living by intervention settings**


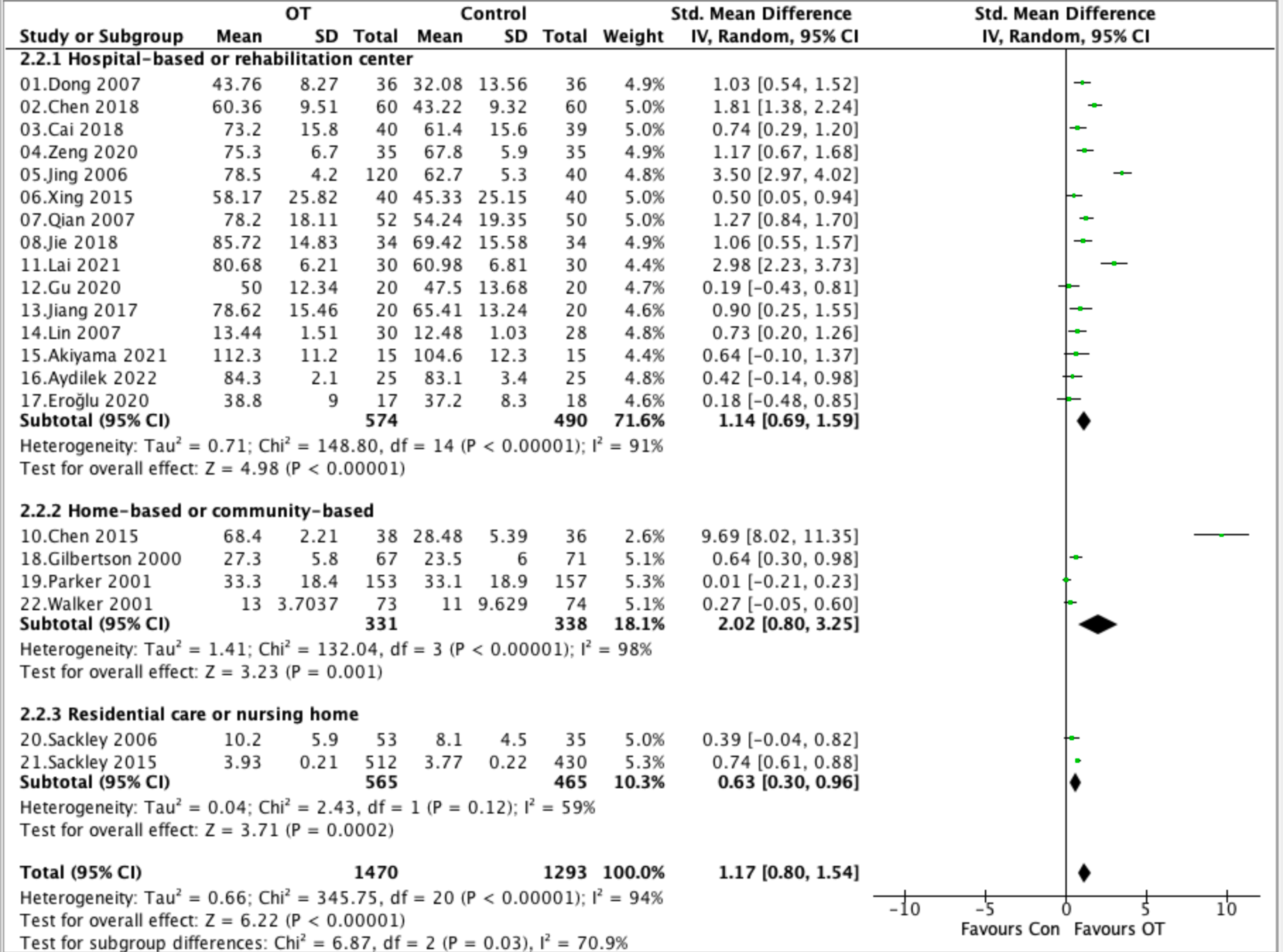


**Figure 4.3. Subgroup analysis of Depressive symptoms by intervention settings**


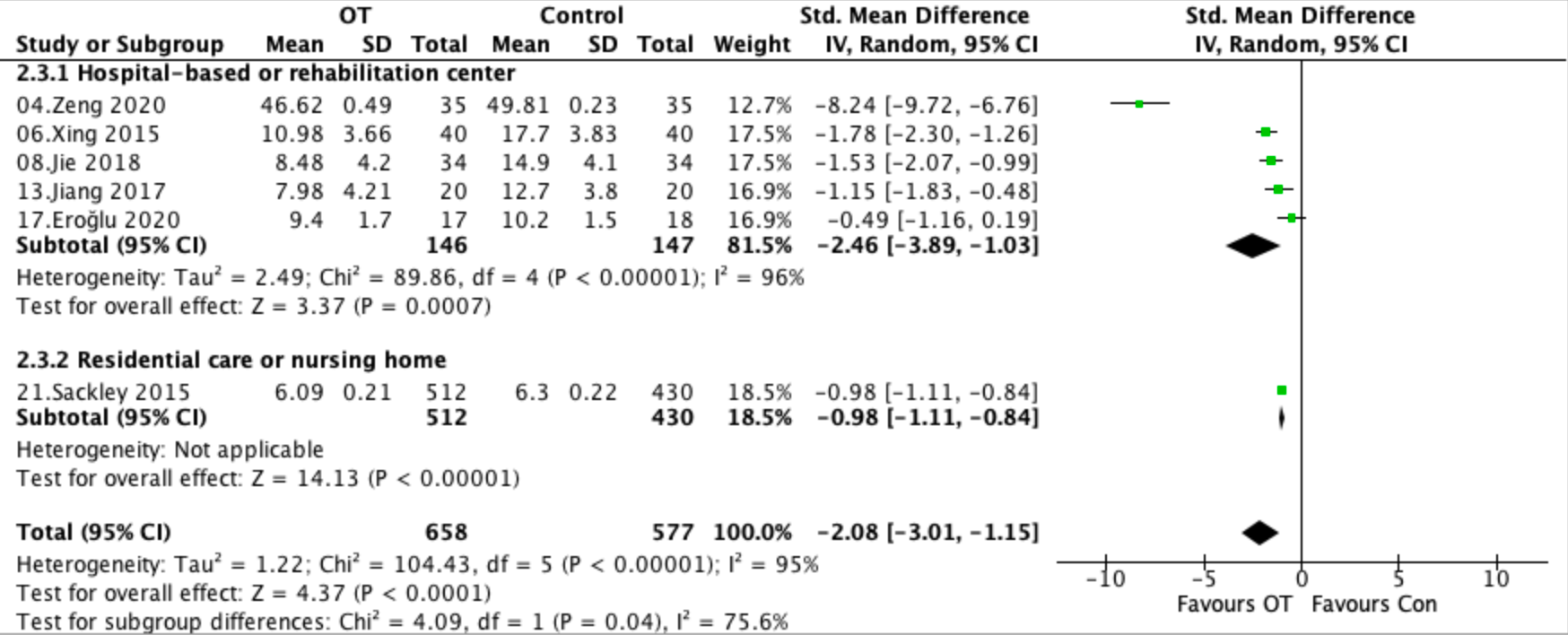


**Figure 4.4. Subgroup analysis of upper limb function by OT Intervention Type**

**
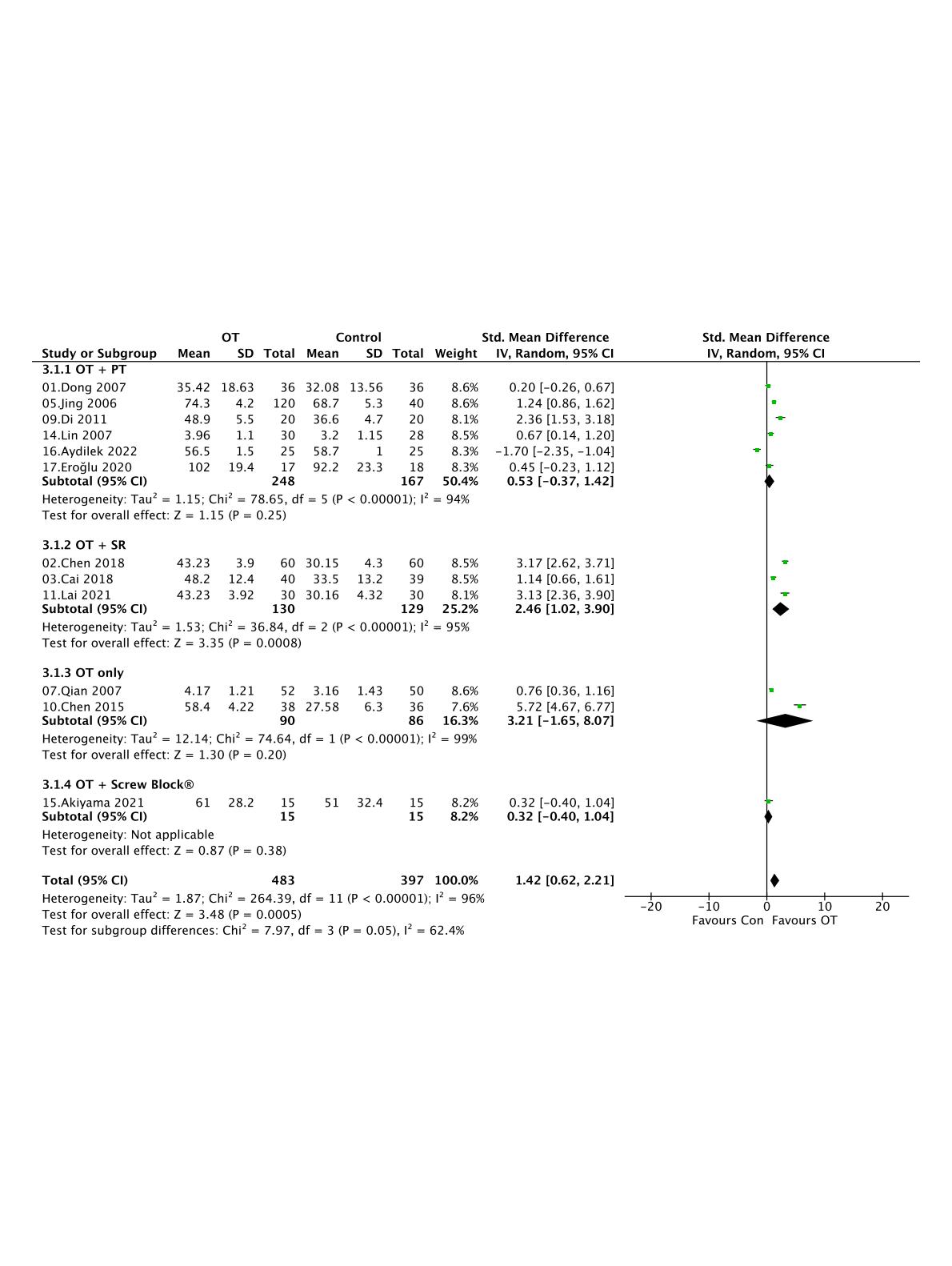
**

**Figure 4.5. Subgroup analysis of Activities of daily living by OT Intervention Type**


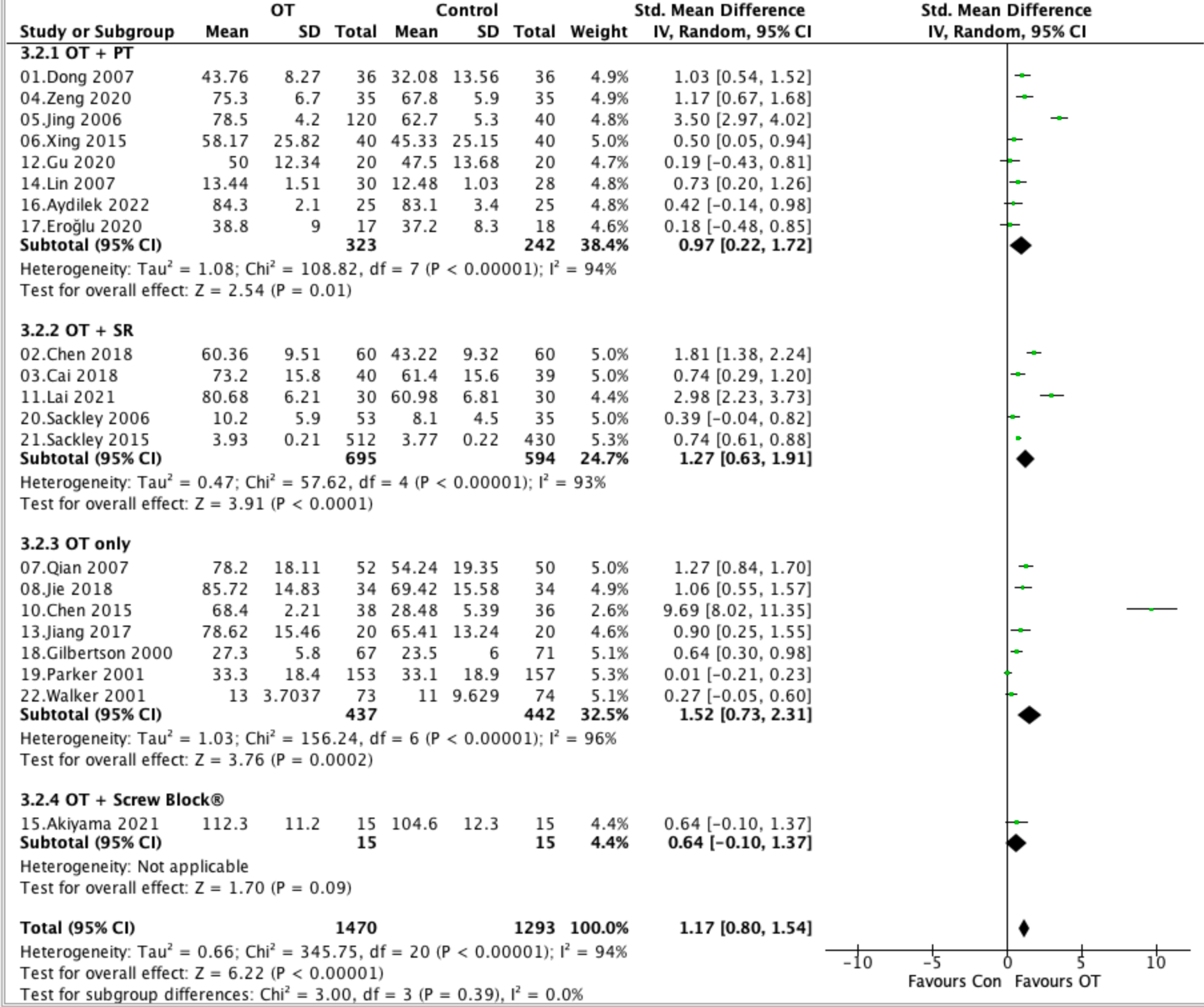


**Figure 4.6. Subgroup analysis of Depressive symptoms by OT Intervention Type**


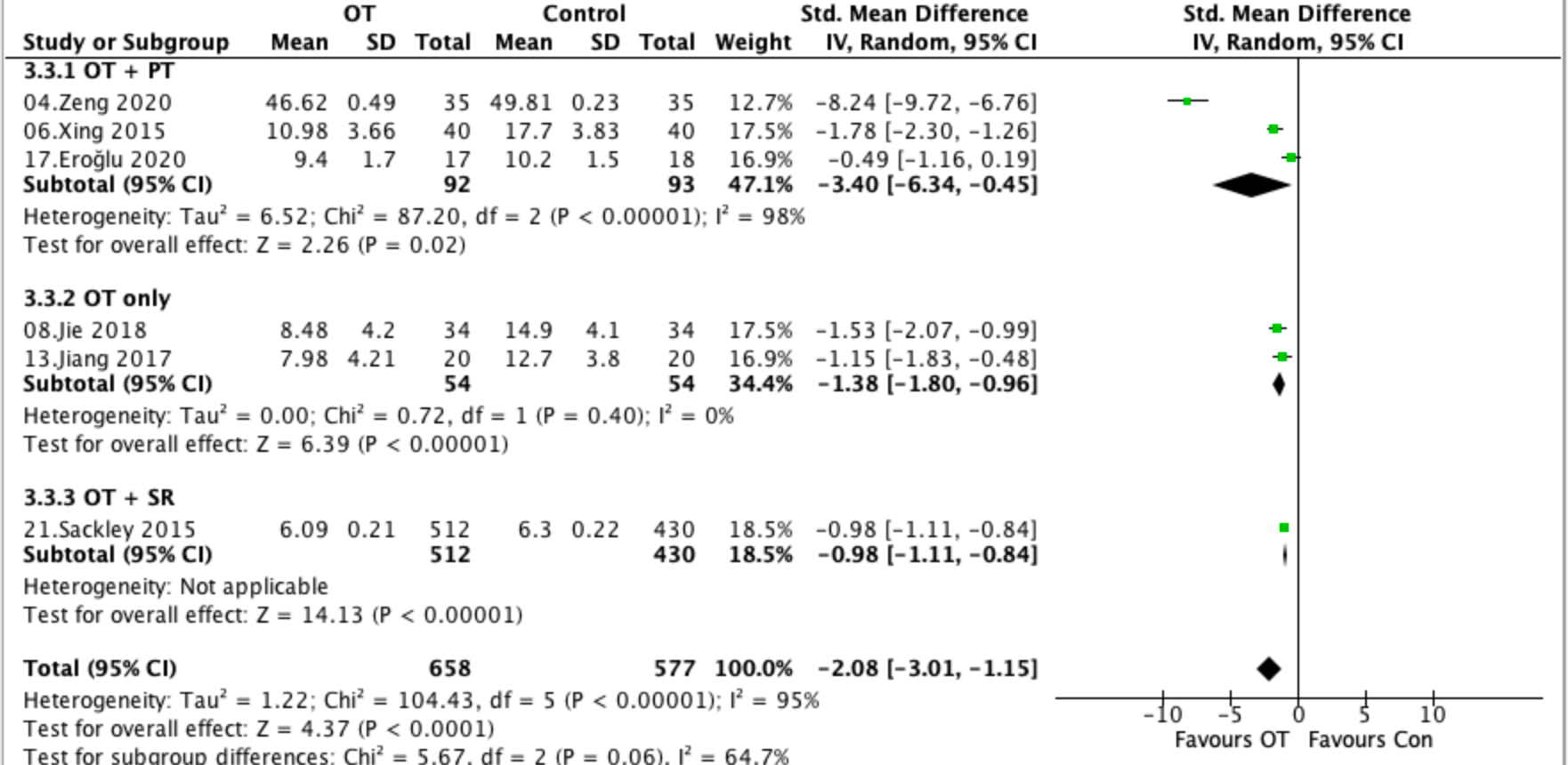


**Figure 4.7. Subgroup analysis of upper limb function by Intervention Duration**

**
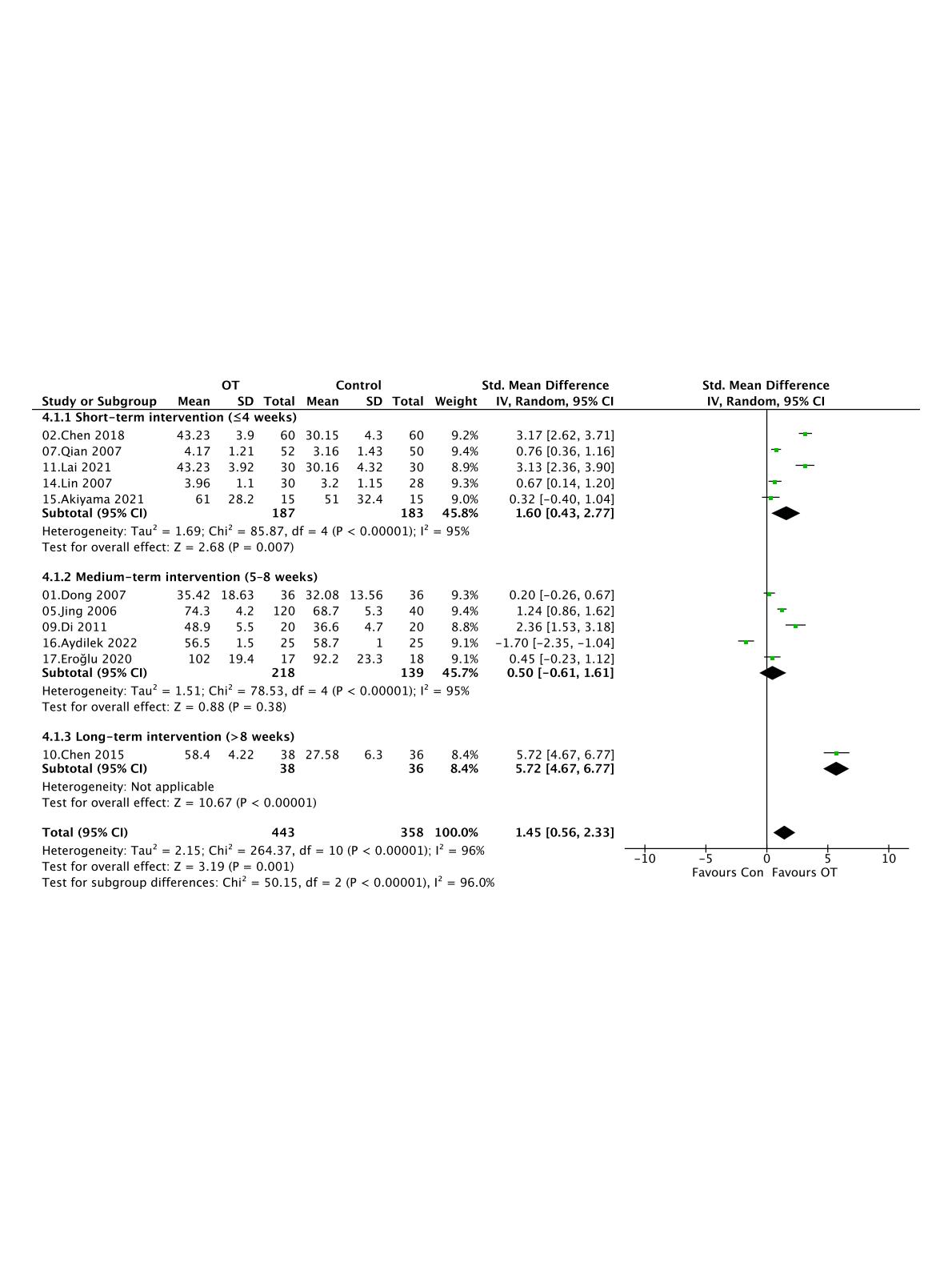
**

**Figure 4.8. Subgroup analysis of Activities of daily living by Intervention Duration**


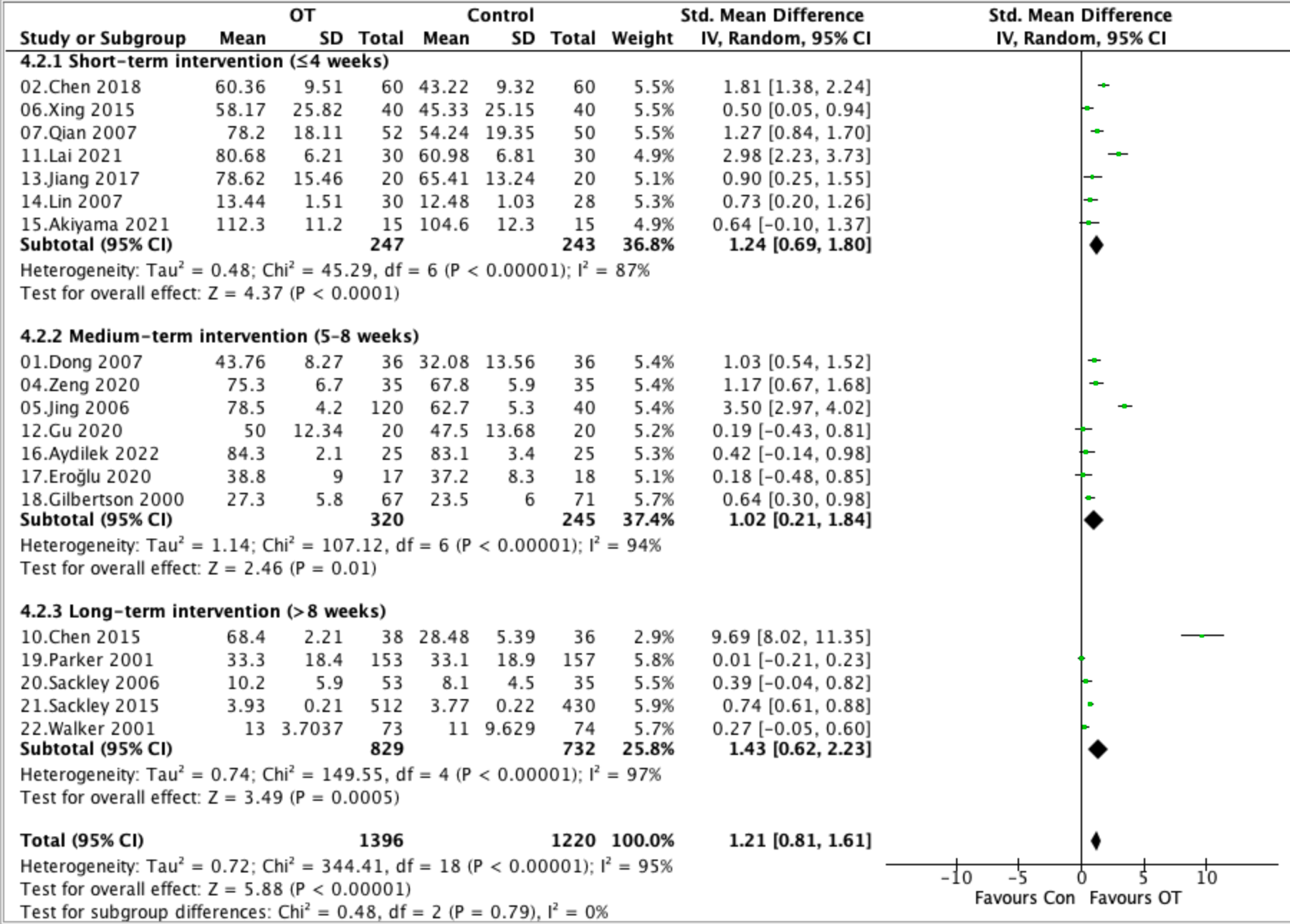


**Figure 4.9. Subgroup analysis of Depressive symptoms by Intervention Duration**


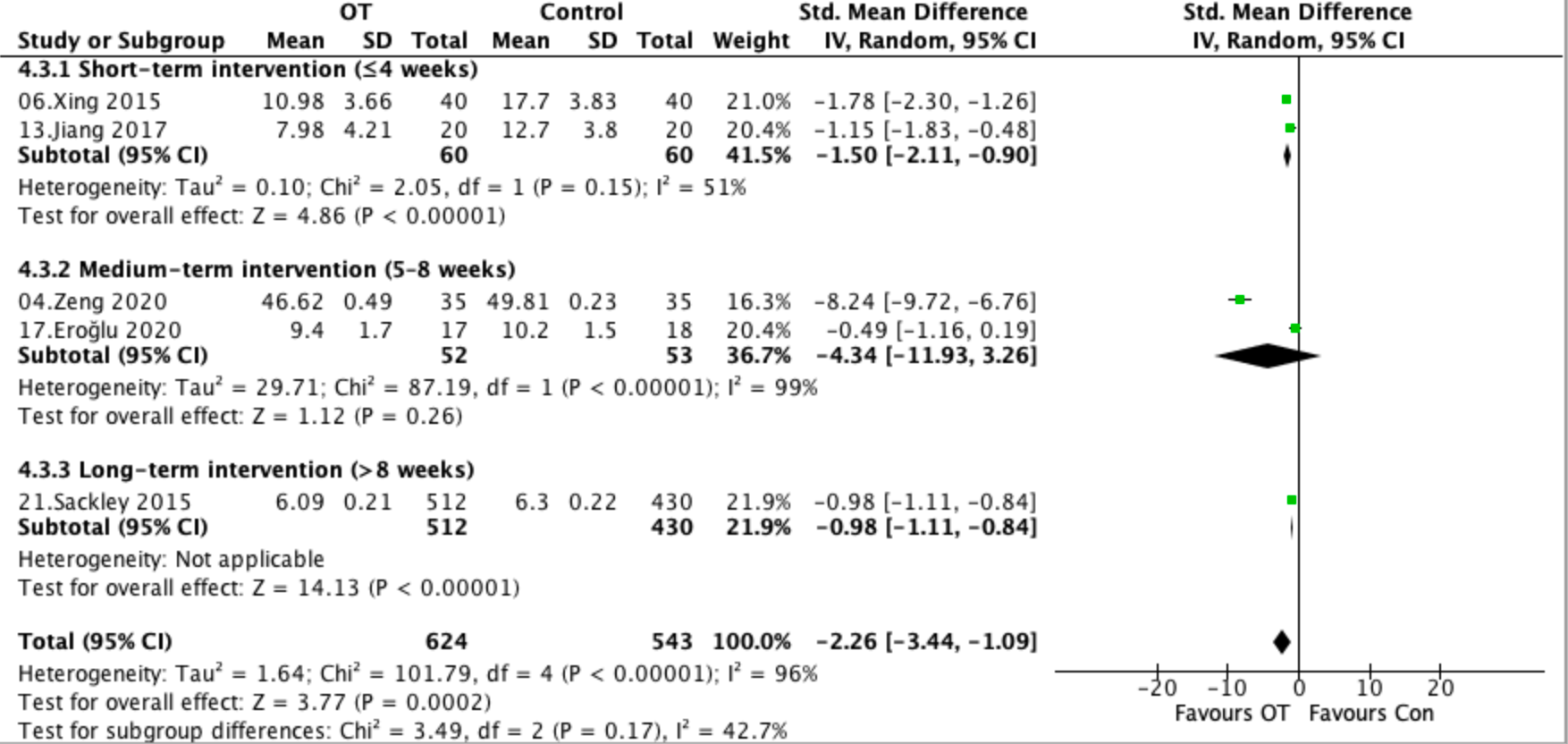


**Appendix 5: Sensitivity analysis**

**1.upper limb function**


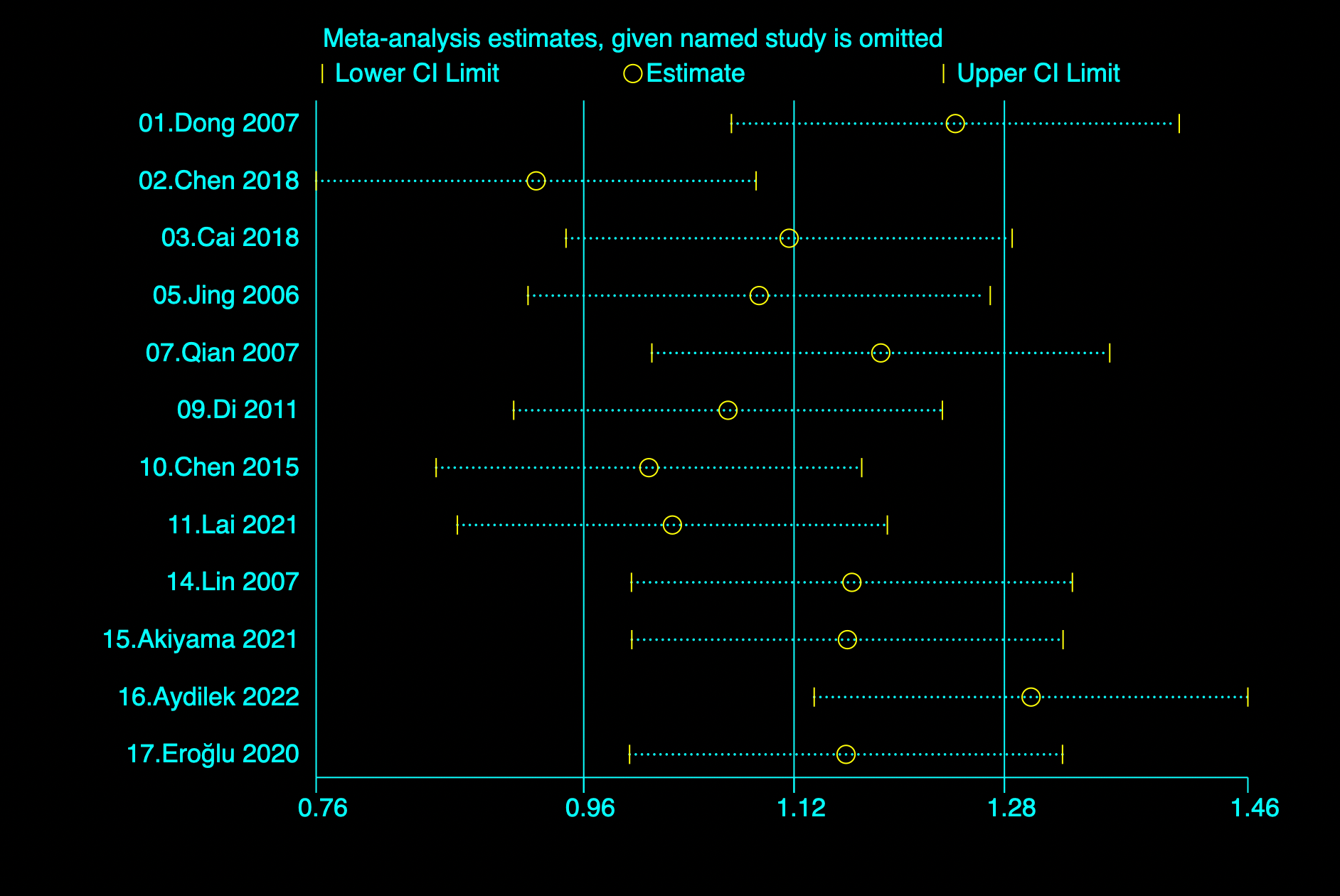


1. Activities of daily living


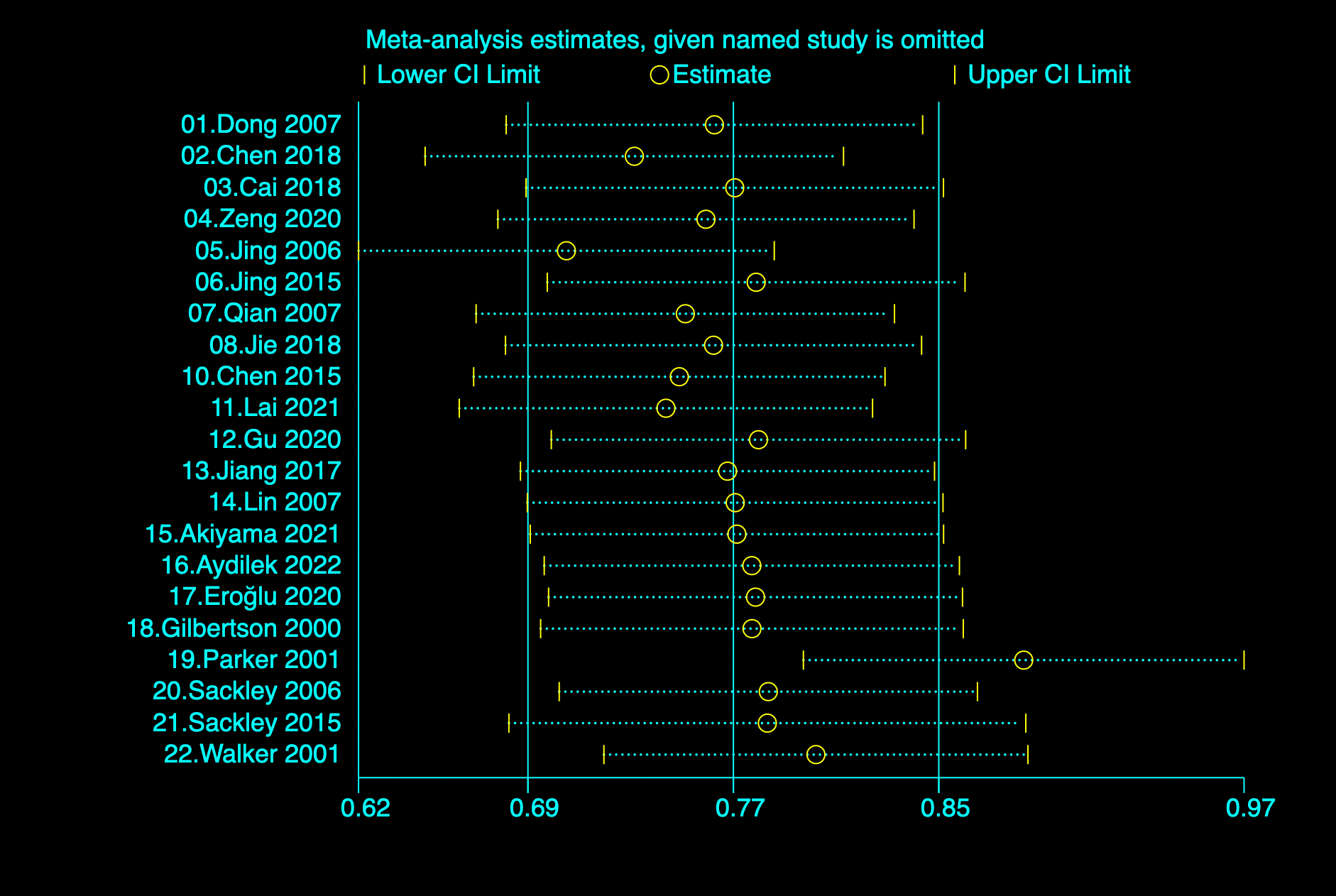


1. Depressive symptoms


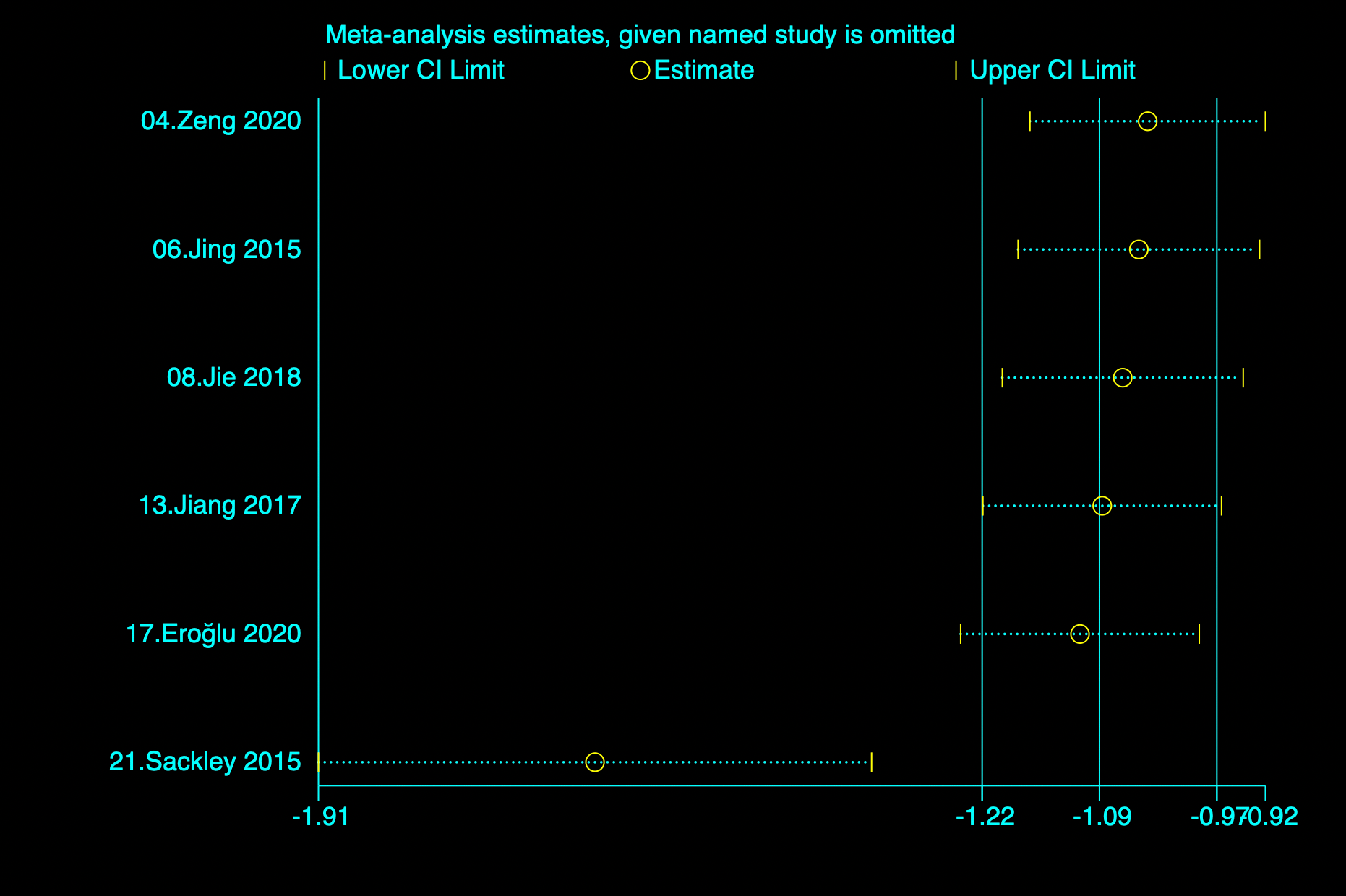


**Appendix 6: Funnel plot**

**1.upper limb function**


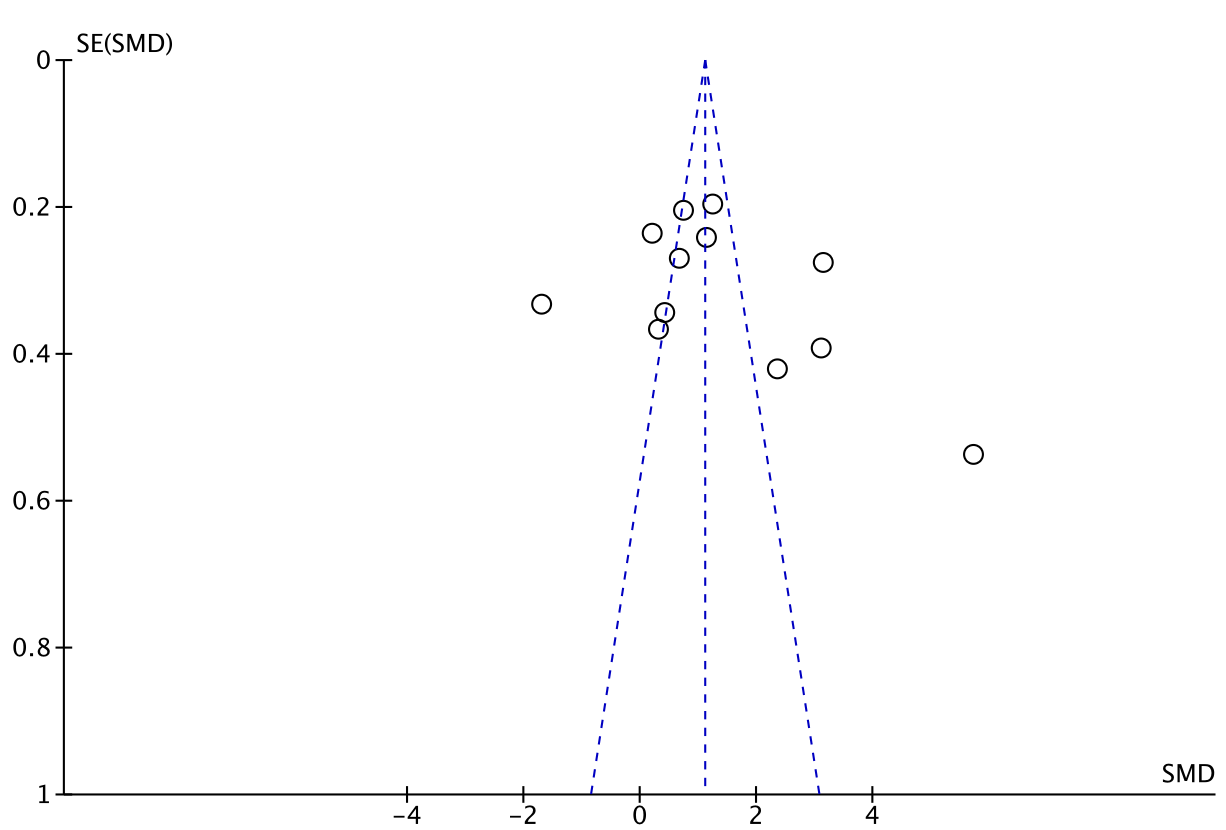


2 Activities of daily living


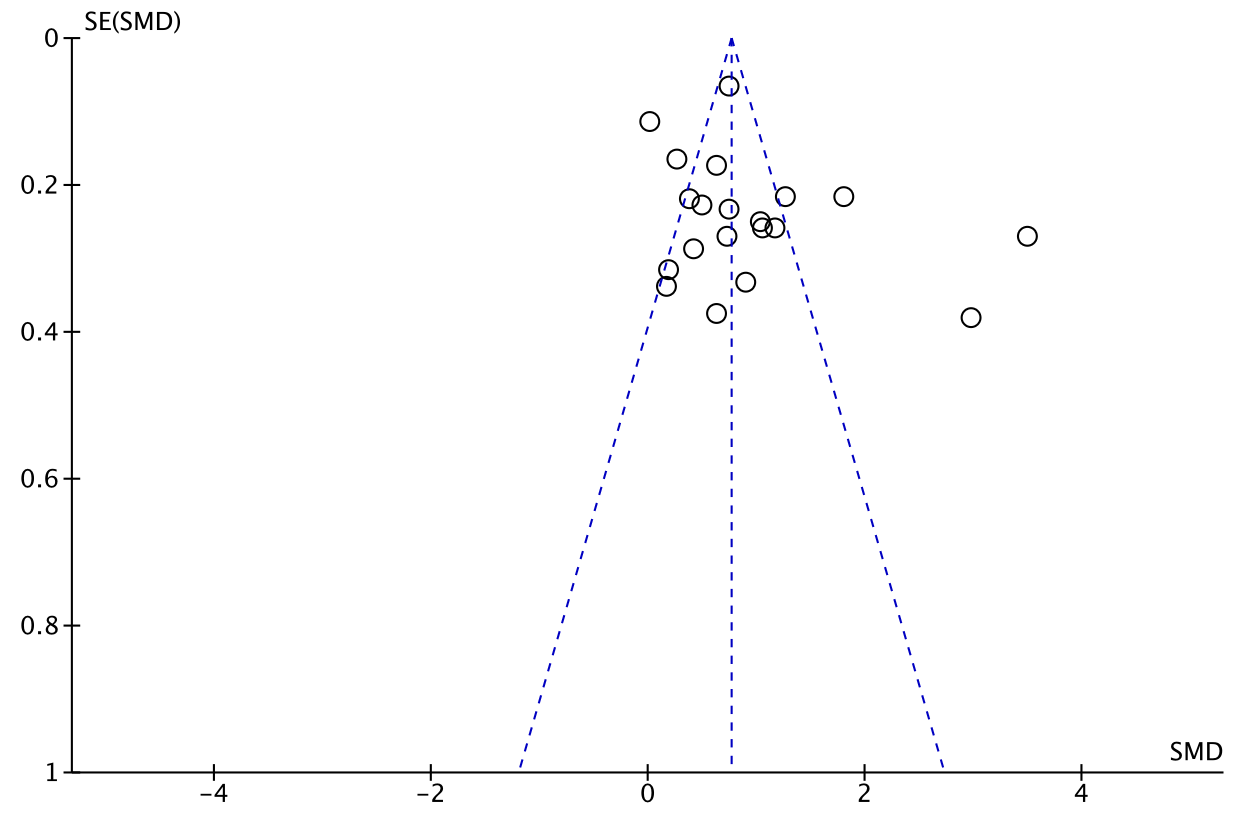


**Appendix 7：GRADE Evidence Profile for All Outcomes**

| **Certainty assessment** | | | | | | | **№ of patients** | | **Effect** | | **Certainty** |
| --- | --- | --- | --- | --- | --- | --- | --- | --- | --- | --- | --- |
| **№ of studies** | **Study design** | **Risk of bias** | **Inconsistency** | **Indirectness** | **Imprecision** | **Other considerations** | **[intervention]** | **[comparison]** | **Relative (95% CI)** | **Absolute (95% CI)** |  |
| 12 | randomised trials | serious | not serious | not serious | not serious | none | 483 | 397 | - | SMD **1.42 higher** (0.62 higher to 2.21 higher) | ⨁⨁◯◯ Low |
| 21 | randomised trials | serious | not serious | not serious | not serious | none | 1470 | 1293 | - | SMD **1.17 higher** (0.8 higher to 1.54 higher) | ⨁⨁◯◯ Low |
| 6 | randomised trials | serious | not serious | not serious | not serious | none | 658 | 577 | - | SMD **2.08 lower** (3.01 lower to 1.15 lower) | ⨁⨁◯◯ Low |

**Appendix 8：Meta regression**

| **Factors** | **Upper limb function** | | | **ADL** | | | **Depression** | | |
| --- | --- | --- | --- | --- | --- | --- | --- | --- | --- |
|  | Coefficient | 95% CI | P value | Coefficient | 95% CI | P value | Coefficient | 95% CI | P value |
| Mean age | -0.01 | -0.05, 0.03 | 0.62 | -0.02 | -0.07, 0.03 | 0.41 | 0.01 | -0.04, 0.06 | 0.74 |
| Dose intensity (h/week) | 0.04 | -0.02, 0.10 | 0.18 | 0.03 | -0.04, 0.09 | 0.33 | -0.02 | -0.08, 0.04 | 0.49 |
| Setting (hospital vs community) | 0.08 | -0.15, 0.31 | 0.49 | 0.11 | -0.12, 0.34 | 0.36 | 0.05 | -0.18, 0.28 | 0.67 |
| Follow-up duration (weeks) | 0.002 | -0.01, 0.01 | 0.72 | 0.003 | -0.01, 0.02 | 0.58 | -0.004 | -0.02, 0.01 | 0.54 |
